# Predicting structural material degradation in advanced nuclear reactors with ion irradiation

**DOI:** 10.1038/s41598-021-82512-w

**Published:** 2021-02-03

**Authors:** Stephen Taller, Gerrit VanCoevering, Brian D. Wirth, Gary S. Was

**Affiliations:** 1 Department of Nuclear Engineering and Radiological Sciences, University of Michigan, Ann Arbor, MI USA; 2 Nuclear Energy and Fuel Cycle Division, Oak Ridge National Laboratory, P.O. Box 2008, Oak Ridge, TN 37831 USA; 3 Nuclear Engineering, University of Tennessee–Knoxville, Knoxville, TN USA

**Keywords:** Structural materials, Nuclear fusion and fission, Computational methods

## Abstract

Swelling associated with the formation and growth of cavities is among the most damaging of radiation-induced degradation modes for structural materials in advanced nuclear reactor concepts. Ion irradiation has emerged as the only practical option to rapidly assess swelling in candidate materials. For decades, researchers have tried to simulate the harsh environment in a nuclear reactor in the laboratory at an accelerated rate. Here we present the first case in which swelling in a candidate alloy irradiated ~ 2 years in a nuclear reactor was replicated using dual ion irradiation in ~ 1 day with precise control over damage rate, helium injection rate, and temperature and utilize physical models to predict the effects of radiation in reactors. The capability to predict and replicate the complex processes surrounding cavity nucleation and growth across many decades of radiation dose rate highlights the potential of accelerated radiation damage experiments. More importantly, it demonstrates the capability to predict the swelling evolution and the possibility to predict other features of the irradiated microstructure evolution that control material property degradation required to accelerate the development of new, radiation-tolerant materials.

## Introduction

The continued contribution of nuclear power to carbon-free electricity generation will require the development of more efficient advanced reactors while maintaining safety and security. These advanced reactor designs impose harsher environments of higher temperatures and more intense radiation fields than current light water reactors and necessitate accelerated materials development to ensure components withstand radiation-induced degradation^1^. Reactor irradiation campaigns to understand radiation effects on microstructure and properties take years to complete, are extremely expensive, and hampered by the paucity of test reactors, all of which contribute to the historical problem of a glacial pace of research to assess candidate materials. For decades, researchers, individual laboratories and international organizations^2–11^ have been searching for methods and theory to accelerate radiation damage in structural materials for nuclear energy applications to speed the development of new, more radiation resistant materials. Ion irradiation offers the capability to shrink the irradiation time from many years in a reactor to days using an accelerated damage rate to predict microstructure and mechanical property changes at lower cost^9,12,13^. Therefore, ion irradiation is the only viable means to study many damaging degradation processes that could limit component lifetime, such as irradiation-induced swelling.

The dimensional instability from swelling can cause operational tolerances to be quickly exceeded and arises from cavities in irradiated materials^14^ as observed and the subject of further research^5,15–18^. Cavity nucleation depends on the coalescence of defects generated from irradiation damage. The generation of gases by transmutation, such as helium, stabilizes cavity embryos and results in bubble formation, further complicating the nucleation process, which also depends on the crystal structure and alloy microstructure. Ion irradiation can simulate the reactor environment by creating damage with self-ions while simultaneously injecting helium as a surrogate for transmutation. The high damage rates (quantified as displacements per atom per second, dpa/s) accessible with ion irradiation (10^–4^ to 10^–3^ dpa/s) compresses the irradiation time by about three orders of magnitude. Yet for accelerated irradiations to be a useful tool for studying and qualifying nuclear materials, the roles and interdependencies of damage rate, helium content, and irradiation temperature on cavity nucleation and growth, along with the radiation-damaged microstructure, must be understood predictively.

**Figure 1:**
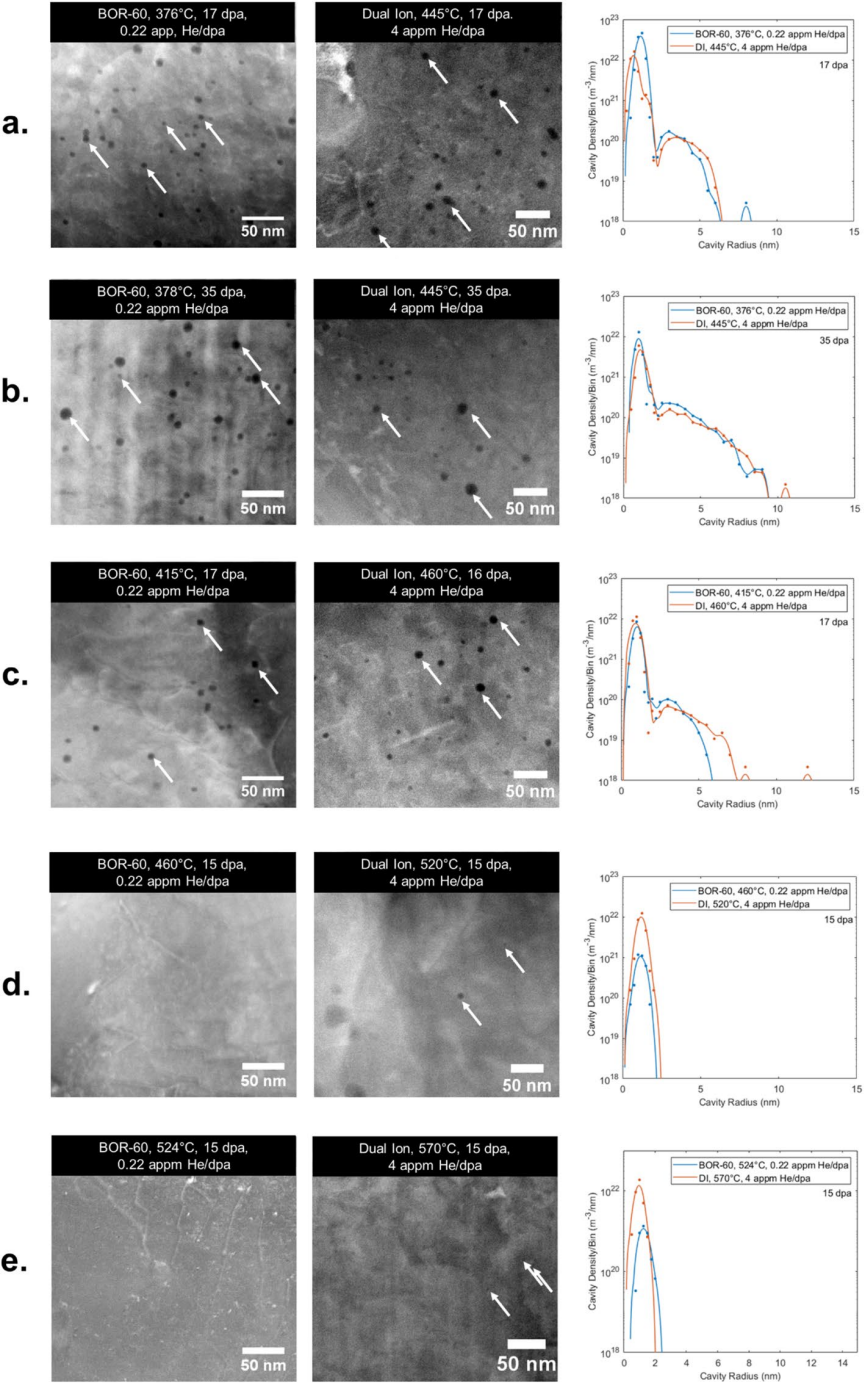
A comparison of scanning transmission electron microscopy–high-angle annular dark field (STEM-HAADF) images of T91 from BOR-60 irradiation^19^ and complementary dual ion irradiations^20^ with corresponding cavity size distributions showing the high degree of agreement. Damage rate is 6–9 × 10^−7^ dpa/s for BOR-60 irradiations and 5–8 × 10^–4^ dpa/s plus injection of 4 appm He/dpa for ion irradiations. The arrows highlight several observed cavities. Details on how the cavity size distributions were obtained are available in Ref.^19–21^ and summarized in the Supplemental Materials for this article.

This paper identifies the underlying processes governing cavity nucleation and early stage growth in a ferritic-martensitic steel, T91, neutron irradiated in the BOR-60 reactor^19^ at a dose rate between 6 × 10^−7^ dpa/s and 9 × 10^−7^ dpa/s to doses between 15 and 35 dpa at 376–524 °C and applies that understanding to successfully replicate the cavity microstructures with dual ion irradiation^20^. Systematic dual ion irradiations^20,21^ that complement reactor irradiations^19^ were performed with 5 MeV iron ions at about 7 × 10^−4^ dpa/s (1.7 × 10^16^ Fe^2+^ ions m^−2^ s^−1^) to doses between 15 and 35 dpa at 445 °C–570°C with 4 appm He/dpa co-implanted simultaneously. The reactor and ion irradiations were coupled with computational modeling of the point defect kinetics and helium trapping to identify the processes that produce equivalent cavity microstructures in reactor and dual ion–irradiated T91 steel. The systematic ion irradiation and modeling produced a microstructure match between the ion and neutron irradiations by increasing the irradiation temperature and the helium injection rate, extending the temperature shift approach initially proposed by Mansur^4^. Figure 1 shows the cavity size distributions for several temperatures and damage levels measured following dual ion (DI) irradiation designed to match those from irradiation in the BOR-60 reactor. Qualitatively, both small and large cavities were observed in a bimodal distribution and transitioned to a unimodal distribution at high temperatures with a consistent shift in temperature (+ 70 °C). Quantitively, the distributions match in both number density and cavity radius, stemming from an increase in the helium injection rate (~ 18 ×) and a strongly accelerated damage rate (~ 10^3^ ×). The interactions of these key parameters will be discussed in detail later to demonstrate the understanding of how reactor microstructures were emulated in the accelerator laboratory. The understanding of how to achieve agreement establishes the utility of ion irradiation as a predictive tool for reactor-irradiated microstructures and paves the way for accelerated materials development for advanced nuclear reactor designs.

## Understanding the cavity size distribution in reactor irradiations

Many aspects of cavity formation have been modeled using the cavity growth rate equation for a homogenous medium^22,23^:

1 $$\frac{dr}{{dt}} = \frac{\Omega }{r}\left[ {D_{v} C_{v} - D_{i} C_{i} - D_{v} C_{v,T} \exp \left( {\frac{2\gamma }{r} - p_{g} } \right)} \right]$$

Damage rate, helium injection rate, and temperature interact with the growth of a cavity of radius $r$ (m) through the net absorption of vacancies at cavities ($D_{v} C_{v} - D_{i} C_{i}$) and the thermal emission of vacancies that is offset by the helium pressure inside the cavities ($D_{v} C_{v,T} {\text{exp}}\left( {\frac{2\gamma }{r} - p_{g} } \right)$), where $D$ is the diffusion coefficient of interstitials or vacancies (m^2^ s^−1^), $C$ is the concentration of each specie, γ is the surface energy (Jm^−2^), $p_{g}$ is the pressure of helium (Pa) and Ω is the atomic volume (m^3^). Point defect concentrations can be calculated using a set of rate equations that balance the defect production rate ($G_{0}$, in the same units as $C_{v}$ and $C_{i}$) against the loss of defects to mutual annihilation with a reaction rate *R*_*iv*_ and to sinks (defined by their sink strength k^2^ in m^-2^):

2 $$\frac{{dC_{{\left( {i,v} \right)}} }}{dt} = 0 = G_{0} - R_{iv} C_{i} \left( {C_{v} + C_{v,T} } \right) - k_{{\left( {i,v} \right)}}^{2} D_{{\left( {i,v} \right)}} C_{(i,v)}$$

Further explanation and definitions of terms used to calculate the cavity growth rate and point defect concentrations are described in the Methods section. This relatively simple set of equations allows for a rough prediction of cavity nucleation in the BOR-60 irradiations and depends heavily on the irradiated microstructure, which is fully characterized and presented in Methods.

**Figure 2:**
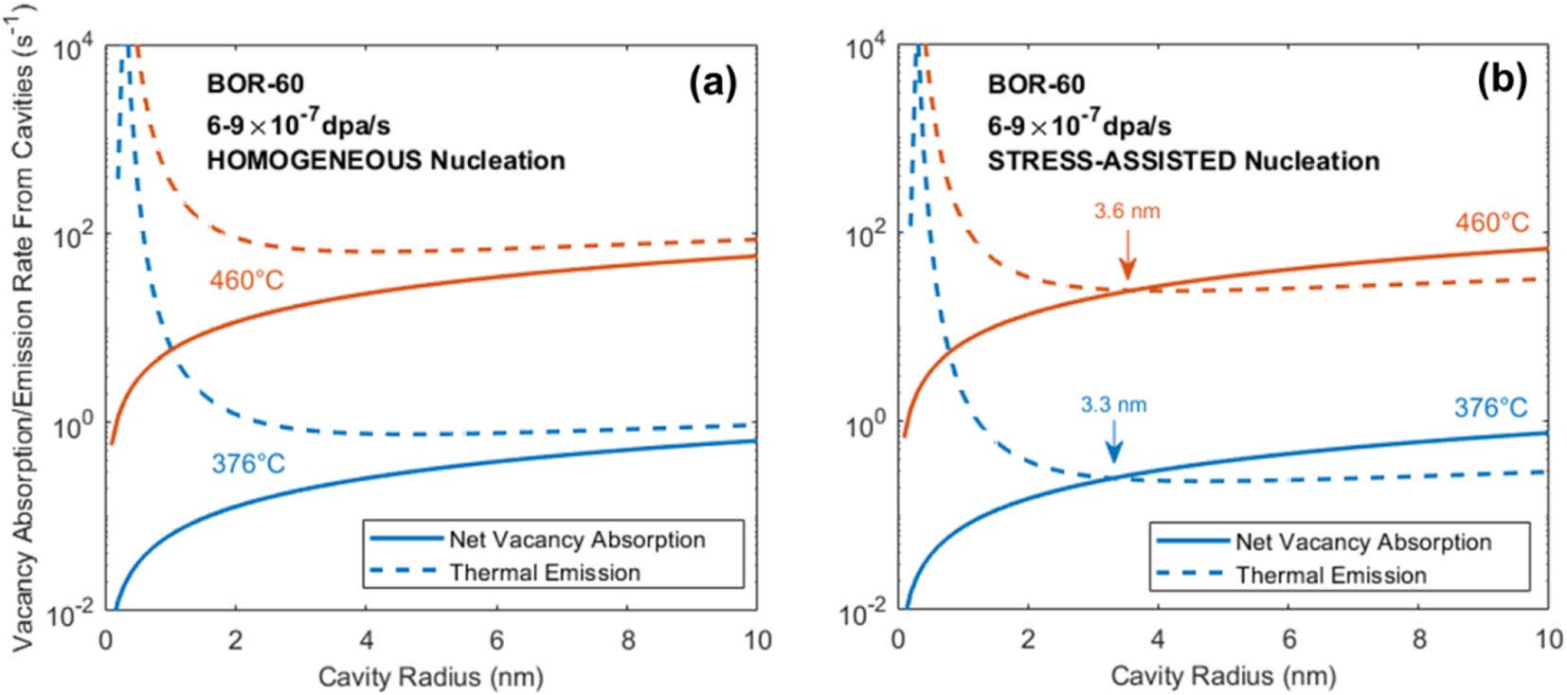
Comparison of the net vacancy absorption and thermal vacancy emission components of cavity growth for BOR-60 irradiation assuming (**a**) homogeneous nucleation and (**b**) the inclusion of a stress-assisted mechanism.

Figure 2a shows the vacancy absorption and emission rates from cavity nuclei for the BOR-60 reactor irradiation conditions at temperatures of 376 °C and 460 °C. The thermal emission of vacancies exceeds absorption at small cavity radii and indicates that cavities should not be able to nucleate, yet cavities were observed in all neutron irradiations of T91 in the BOR-60 reactor^19^, as characterized using transmission electron microscopy (TEM) and scanning TEM (STEM), as shown in Fig. 1a–c. The small cavities (≤ 2.5 nm in radius) of the bimodal distributions (Fig. 1a–c) are presumed to be helium-filled bubbles, while the larger cavities are presumed to contain some helium but are above a critical radius, enabling bias-driven growth. These larger cavities are henceforth referred to as voids to distinguish them from the bubble population^19^. For cavities to form in the BOR-60 irradiations, there must be one or more mechanisms that either reduce vacancy emission or increase the net absorption of vacancies. Several processes may enhance cavity nucleation. For example, energetic damage cascades may produce cavity embryos directly from the primary damage^24^, or impurities may act to increase the capture efficiency of cavities^17,25^. As well, the dislocation network from the normalization and tempering of T91 and dislocation loops from irradiation cause local stress fields that may modify adjacent point defects concentrations^26,27^. No microstructure model has been able to, or likely can, capture every aspect of microstructural evolution and perform calculations on a reasonable timescale. Therefore, several pragmatic approaches explored in this work have evaluated the importance of nonhomogeneous nucleation sites.

The cavity growth rate equation assumes a homogeneous microstructure with an equal concentration of vacancies throughout the material. The strain field around a nucleation site can be converted to a stress^28^ (σ_rr_) that acts on the cavities and is dependent on the distance from the cavity (r):

3 $$\sigma_{rr} = \frac{E}{{2\pi \left( {1 - \nu } \right)}}\frac{\sin \theta }{r},$$

where E is the elastic modulus, $v$ is Poisson’s ratio, and θ is the angle between the dislocation Burgers vector and the cavity. Further definitions of terms and parameters are provided in the Methods, assuming a density of sites equal to the cavity density in the BOR-60 irradiations. Tensile stress reduces the emission of vacancies by modifying the equilibrium concentration of vacancies at the cavity surface, counteracting the cavity surface energy. Thus, Eq. (1) is modified (following Ref.^26,29^) as

4 $$\frac{dr}{{dt}} = \frac{\Omega }{r}\left[ {D_{v} C_{v} - D_{i} C_{i} - D_{v} C_{v,T} \exp \left( {\frac{2\gamma }{r} - p_{g} - \sigma } \right)} \right].$$

The impact of this stress effect on the emission of vacancies is plotted in Fig. 2b for BOR-60 irradiations and acts to reduce emission terms for all irradiation conditions. Absorption exceeds emission at a radius of 2–4 nm, which would transition helium bubbles to voids, making nucleation possible in reactor dose rate conditions. Thus, an enhanced nucleation mechanism must be occurring to produce the observed cavities. This implies that cavities nucleate at enhanced nucleation sites, and so other possible nucleation-enhancing mechanisms must also be considered. Its impact on the relationship between damage rate and temperature, and the effect of the damage rate on the helium injection rate, will be elaborated on in the next section.

## Predicting the cavity size distributions using ion irradiations

**Figure 3:**
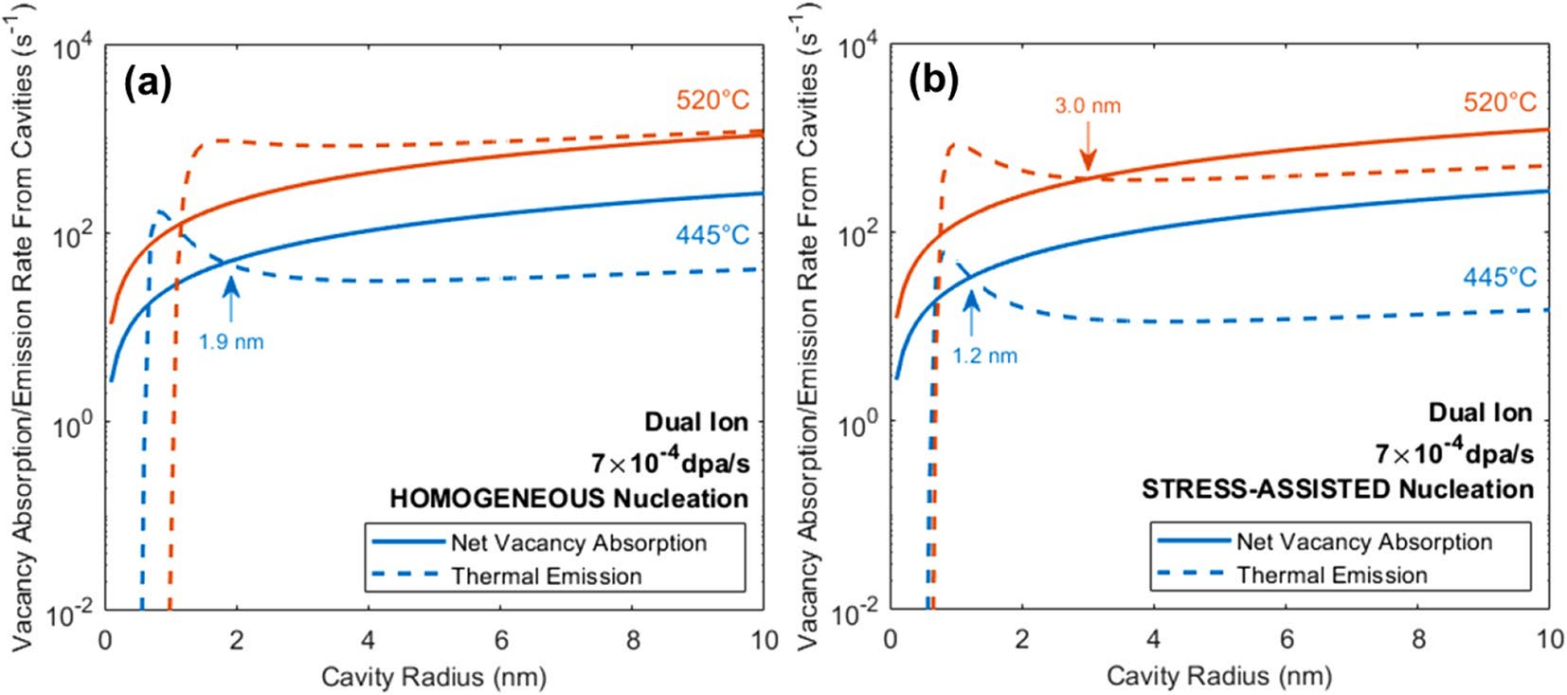
Comparison of the net vacancy absorption and thermal vacancy emission components of cavity growth for dual ion irradiation assuming (**a**) homogeneous nucleation and (**b**) the inclusion of a stress-assisted mechanism.

At high ion irradiation damage rates, both the temperature and helium injection rate required to match the cavity size distribution of reactor irradiations must be assessed. In ion irradiations, the high damage rate increases the vacancy concentration orders of magnitude above that in reactor such that homogenous nucleation is sufficient (Fig. 3a) to enhance net vacancy absorption relative to vacancy emission for cavity sizes of 1–3 nm, indicating the potential for void nucleation at rates similar in magnitude with that for reactor irradiation. With a stress-assisted nucleation mechanism (Fig. 3b), there is very little change to the crossover points at either temperature, indicating that enhanced nucleation may be less pronounced at high damage rates and the transition from bubbles to voids can be explained by homogeneous nucleation.

The relationship between damage rate and temperature will be explored further in this section, first in the context of cavity growth rate and the impact of enhanced nucleation on swelling to show that helium trapping dictates the ion irradiation temperature required to match the cavity microstructure between the two irradiation conditions. Further, the incorporation of helium injection rate and helium partitioning will be discussed to explain why helium must be increased in ion irradiation to match the cavity microstructure in the BOR-60 irradiations, thus providing a formula for prediction of reactor-irradiated microstructures.

### Damage rate–temperature relationship in cavity nucleation

The relationship between damage rate and temperature for cavity growth is believed to be well understood^3–5^. As the damage rate increases, the temperature must be increased to maintain the balance between production and loss of defects to sinks. Mansur et al.^3–5^ developed an invariance relationship between damage rate and temperature for growth-dominated microstructural features that relies solely on changes in the diffusion and point defect concentrations to equate cavity growth rates, but this model does not account for the presence of helium. Applying the Mansur invariance relationship to cavity growth for the irradiation conditions in Fig. 1a yields a temperature shift of + 120 °C to apply to the ion irradiation dose rates. This is based on a relatively simple point defect kinetics model that ignores the complex evolution of defect clusters with damage. Cluster dynamics (CD) models remove this limitation by treating each vacancy or vacancy-helium cluster as a sink that evolves with time according to the kinetics of point defect aggregation and dissociation. This approach allows some simplifying assumptions to be relaxed, most notably that of a static number density of sinks and includes the potential for cavity embryo nucleation in the primary damage cascade. Additional details related to the CD simulations are presented in Methods and supplemental materials.

**Figure 4:**
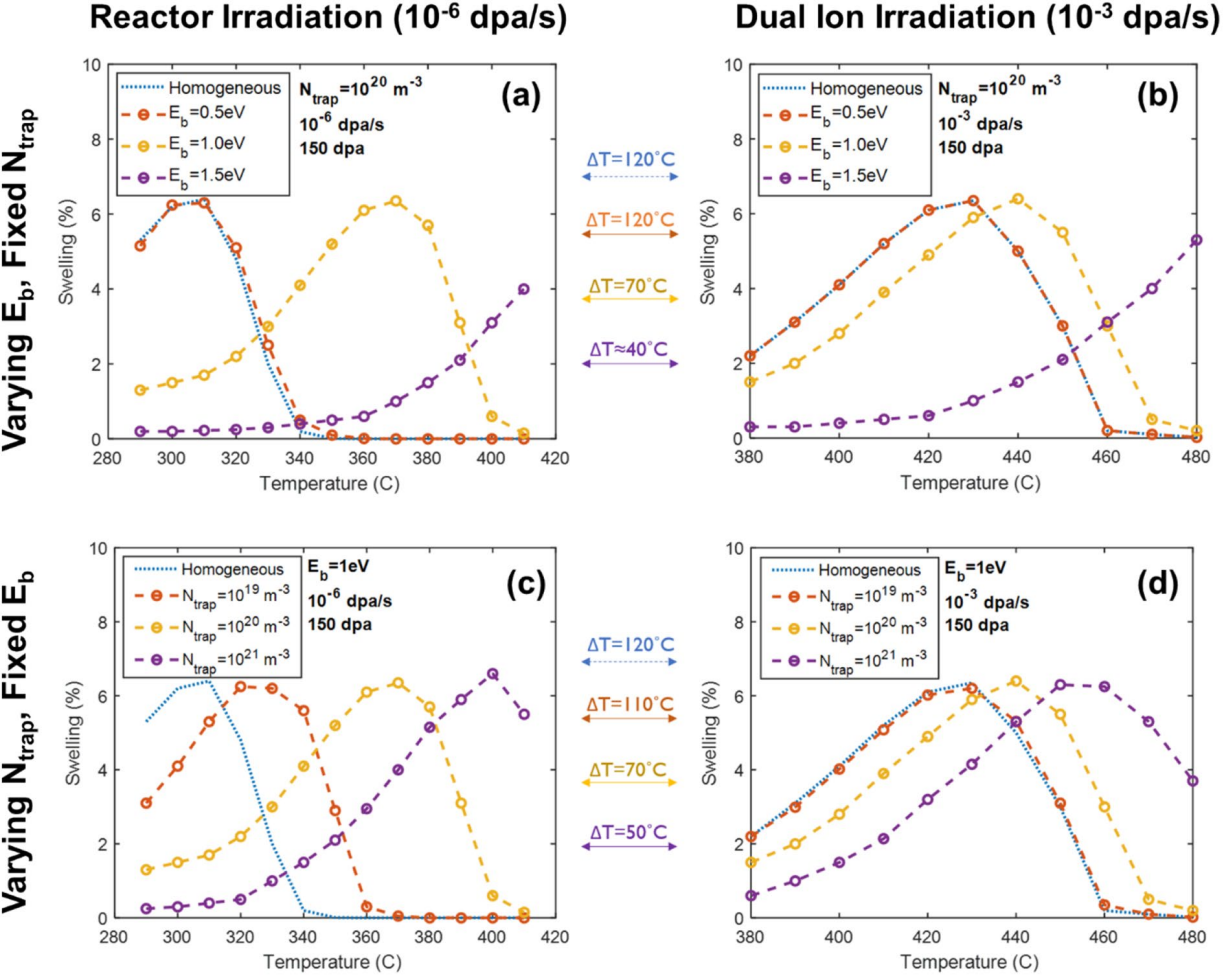
Swelling at 150 dpa as a function of temperature at (**a**) reactor and (**b**) ion irradiation dose rates, generated by a cavity-biased CD simulation for a range of helium-trap binding energies, and the same (**c**) reactor and (**d**) ion irradiation dose rates for a range of trap densities. Even though an increase in either helium binding energy or trap density increases the temperature range over which swelling occurs, the effect is non-linear with increasing damage rate and results in the reduction in the ΔT between reactor and ion irradiation.

A form of enhanced nucleation was implemented in a CD model as trap-assisted nucleation where a constant density of static trapping sites is distributed in the microstructure to immobilize helium, which is then released in proportion to a binding energy. The effect of these sites is twofold: first, the density of traps is independent of temperature and dose rate, effectively providing a lower bound of potential cavity nucleation sites for helium to partition, and second, the de-trapping reaction introduces a temperature dependence, defining the strength at which helium is immobilized by these traps. The effect of this helium trap–assisted versus homogenous nucleation on the peak swelling temperature at each dose rate and trap density is shown in Fig. 4. Note that in these figures, the predicted temperature shift associated with increasing dose rate from 10^−6^ to 10^−3^ dpa/s with only *homogeneous* nucleation is identical to the invariance relationship discussed previously as + 120 °C. Provided a sufficiently high density and binding energy of helium traps, a consistent upward shift in peak swelling temperature is observed. A trap binding energy of 1.0 eV with a density of 10^20^ m^−3^ produces a predicted temperature shift of about + 70 °C.

The enhancement to nucleation is disproportionately strong at low dose rates for which the characteristic time between damage events is long; this allows more thermal emission due to the longer time at temperature. Thus, the peak swelling temperature predicted by homogeneous nucleation at low dose rates will occur at lower temperatures, which have low thermal emission rates. The presence of helium, the partitioning of helium between trapping sites, and the time dependence of helium de-trapping all contribute to a larger effect on predicted swelling at a lower dose rate (reactor) compared with a higher dose rate (ion irradiation). The net result of the helium effects is thus to reduce the temperature shift relative to the classical rate theory (Mansur shift).

The trapping sites provide cavity nucleation sites and are at a density consistent with the experimentally observed void density. Using a helium trap binding energy of 1 eV and an estimated trapping density of 10^20^–10^21^ m^-3^, the CD model results in a predicted temperature shift that is reduced from ~  + 120 °C to about + 70 °C. That is, to match the cavity microstructure of the 376 °C reactor irradiation, the ion irradiation should be conducted at ~ 445 °C, while the reactor irradiation at 460 °C should have an ion irradiation temperature of ~ 530 °C, consistent with the conditions identified in Fig. 1. Thus, the enhancement to nucleation from helium trapping results predicting the shift in temperature with increasing damage rate resulted in the experimentally observed shift. However, this does not determine how much helium needs to be injected for these helium trap nucleation sites, which is addressed in the next section.

### Damage rate–helium injection rate relationship

The helium injection rate used in the dual ion irradiation experiments (4 appm He/dpa) is substantially greater than the transmutation rate estimated in reactor (~ 0.22 appm He/dpa), as noted in Fig. 1. As the damage rate is increased, the amount of helium trapped at sinks other than cavities will increase as helium rapidly partitions to microstructural features with insufficient time to de-trap during the shorter irradiation period^21^. This idea is illustrated using the helium trap and release model described in the Methods and Ref.^21^, which consists of the helium generation (injection), diffusion to a trap, and release from the trap according to the binding energy, and in comparison to a more detailed CD model.

**Figure 5:**
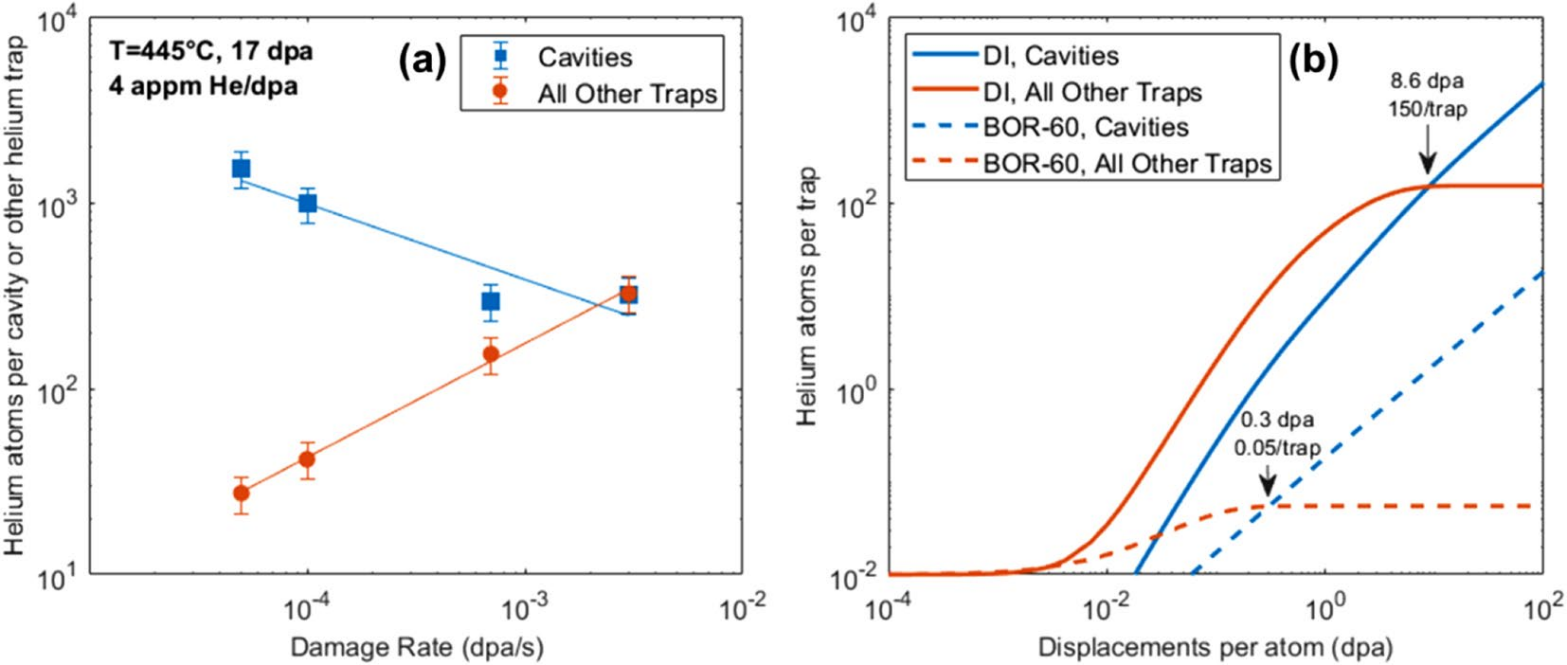
Helium content at cavities and all other traps (dislocation lines, dislocation loops, and HeV clusters) resulting from the trapping/de-trapping process in T91 for (**a**) dual ion irradiation to 17 dpa at 445 °C as a function of damage rate, and (**b**) BOR-60 irradiation at 376 °C with 0.22 appm He/dpa and dual ion irradiation at 445 °C with 4 appm He/dpa as a function of accumulated damage (dpa).

The partitioning of helium among the major traps in the alloy, namely, dislocation lines, dislocation loops, cavities, and helium-vacancy (HeV) clusters, was calculated at 17 dpa as a function of damage rate and helium injection rate, based on the microstructure data presented in Table 1. Figure 5 plots the helium content as a function of damage rate and helium injection rate for cavities and other sinks for the case of dual ion irradiation at 445 °C and 17 dpa with 4 appm He/dpa. The amount of helium trapped at sinks other than cavities increases with damage rate, which reduces the helium available to interact with (trap at) cavities, and thus the effective helium per cavity is a function of the injection rate (Fig. 5a). In this alloy, the dislocation line density is high, but with a relatively low binding energy of 1.0 eV^30^ compared with dislocation loops (2.3 eV^31^), HeV clusters (~ 2.3 eV^32^), and cavities (3.3 eV^33^), and thus the dislocation lines trap very few helium atoms before a steady state condition. As discussed in Taller and Was^21^, helium will trap in proportion to the sink strength of a feature and over time will flow from weaker traps to stronger traps based on the helium binding energy. In both reactor and ion irradiation, other traps besides cavities gather helium quickly. However, the longer time between damage events at the lower reactor irradiation dose rates allows the helium to effectively repartition to the strongest traps during irradiation, leading to relatively quick saturation of lower binding energy traps, as shown in Fig. 5b. Thus, for doses above about 0.5 dpa, helium is primarily trapped at cavities in reactor. However, due to the short irradiation time in ion irradiation experiments, saturation of traps other than cavities does not occur until a larger dose of about 8.6 dpa.

**Table 1:** Summary of characterization results for cavities and dislocation loops in irradiated T91 to use as input parameters. Error in number densities (not shown) is due to TEM foil thickness measurement and is estimated to be 10%. Error in swelling (not shown) is estimated to be about 27% from standard error propagation techniques. N.O. means the feature was not observed. Negl. means the feature was observed but not found in a sufficient amount to form statistically significant values.

T (°C)	Irradiation conditions				Cavities				Dislocation loops		References
	Irradiating particle	Displacement damage (dpa)	Damage rate (10^−4^ dpa/s)	Helium Co-injection Rate (appm He/dpa)	Number density of bubbles (10^20^ m^-3^)	Number density of voids (10^20^ m^-3^)	Swelling (%)	Cavity Sink Strength (10^14^ m^-2^)	Number density of dislocation loops (10^21^ m^−3^)	Dislocation loop line density and sink strength (10^14^ m^−2^)	
376	Neutrons (BOR-60)	17.1	~ 0.008	0.22	1200	7.3	0.016	16.4	2.0	2.0	^19^
445	Fe^2+^ + He^2+^	16.6	7.1	4	178	5.4	0.012	2.3	3.5	2.5	^20^
415	Neutrons (BOR-60)	18.6	~ 0.008	0.22	160	6.5	0.006	2.6	2.0	1.6	^19^
460	Fe^2+^ + He^2+^	16.6	7.5	4	120	5.8	0.017	1.8	1.6	1.8	^20^
460	Neutrons (BOR-60)	14.6	~ 0.007	0.22	32	N.O	N.O	0.5	Negl	Negl	^19^
520	Fe^2+^ + He^2+^	14.6	6.5	4	140	Negl	Negl	2.3	Negl	Negl	^19^
524	Neutrons (BOR-60)	15.4	~ 0.007	0.22	33	N.O	N.O	0.6	N.O	N.O	^19^
570	Fe^2+^ + He^2+^	15.4	6.7	4	170	N.O	N.O	2.3	N.O	N.O	^20^
378	Neutrons (BOR-60)	35	~ 0.008	0.22	220	14.7	0.033	3.8	1.9	1.3	^19^
445	Fe^2+^ + He^2+^	35.0	7.6	4	140	9.4	0.036	2.7	2.8	2.1	^20^

**Figure 6:**
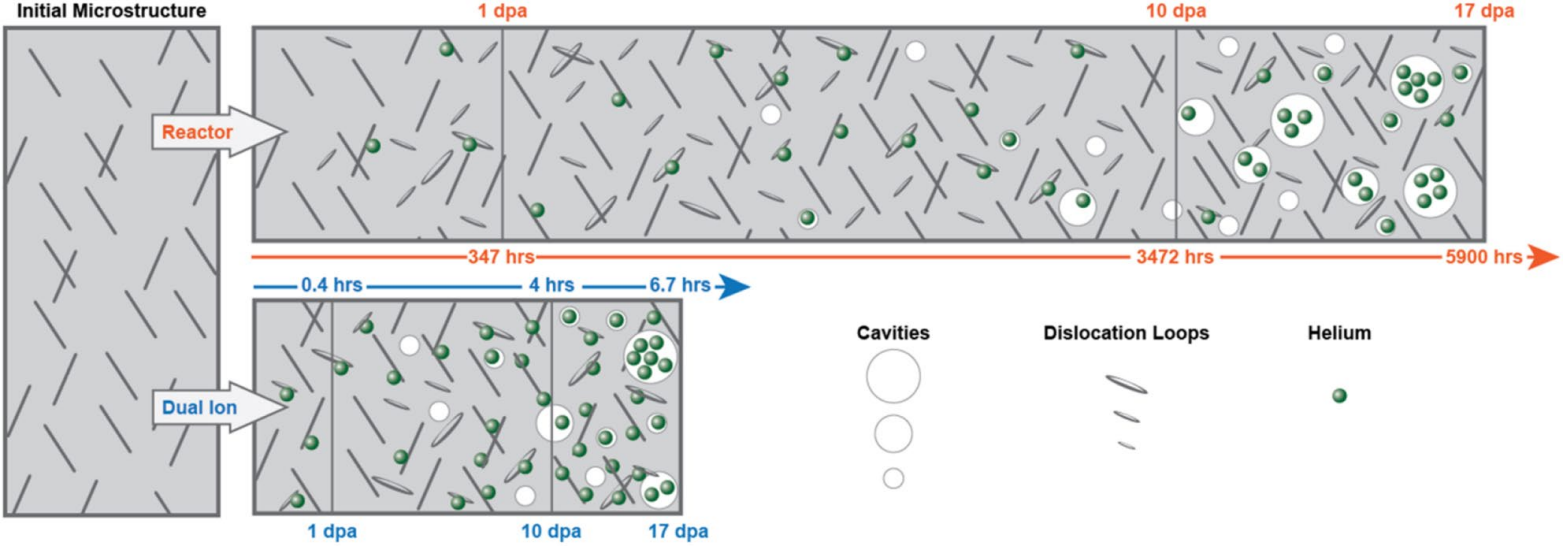
Schematic of the time-dependent helium trapping and release behavior under reactor and ion irradiation conditions. With the higher damage rate of dual ion irradiation compared to reactor irradiation, more helium becomes trapped at microstructural features other than cavities requiring additional helium to be injected.

The behavior of non-cavity traps can be described as consisting of three time-based regimes: buildup of helium in the initial microstructure (dislocation lines) and primary damage clusters (HeV clusters); buildup at dislocation loops until a steady-state is reached; and continued release from all of these traps and partitioning to cavities. In both reactor and ion irradiation, buildup in the initial microstructure dominates the relative partitioning of helium until about 0.005 dpa (about 2 h in reactor and about 7 s in ion irradiation). At this point, dislocation loops are nucleating and also trapping helium. Because of the lack of time to de-trap from dislocation loops at the higher dose rate of ion irradiation, the flow of helium from dislocation loops to cavities occurs much later in dose compared with reactor irradiation, as schematically illustrated in Fig. 6. Therefore, the helium injection rate must be increased with an increase in damage rate to compensate for the insufficient time for helium release from weaker traps and flow to cavities.

**Figure 7:**
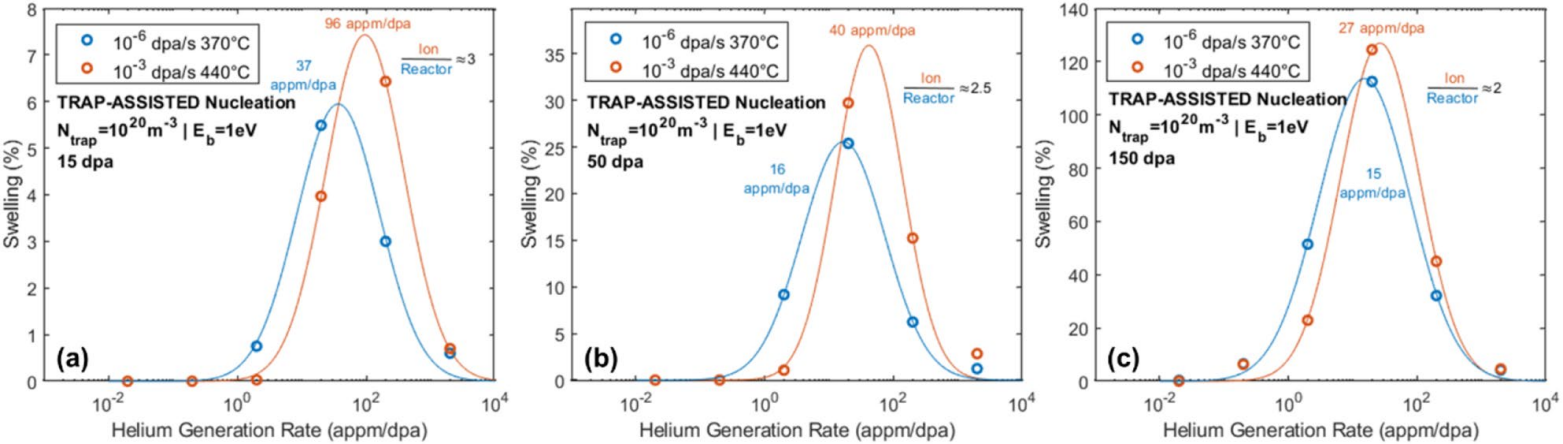
Swelling at (**a**) 15 dpa, (**b**) 50 dpa, and (**c**) 150 dpa as a function of helium co-injection rate for ion (10^−3^ dpa/s) and fast reactor (10^–6^ dpa/s) dose rates with helium traps with *E*_b_ = 1 eV and density of 10^20^ m^-3^.

With the previous determinations of the impact of helium trappings sites and helium partitioning among these sites, CD was used to calculate swelling as a function of helium generation rate using trap-assisted nucleation for different damage levels and temperatures corresponding to the reactor and dual ion irradiations, as presented in Fig. 7. The magnitudes and shapes of the swelling curves are very similar, confirming the + 70 °C temperature increment used for ion irradiation with helium traps configured with a binding energy of 1 eV and a density of 10^20^ m^-3^. However, the consistent upward shift in peak swelling of the helium generation rate for the high-dose-rate conditions indicates that ion irradiation at a high damage rate requires co-injected helium at rates ~ 10 × higher than in reactor to simulate reactor swelling. The selection of 4 appm He/dpa in the experiments described in Fig. 1 confirms the roughly order-of-magnitude-greater helium injection rate needed in high-dose-rate irradiations to match the cavity size distribution in reactor at ~ 15 and 35 dpa. The time-dependent nature of the swelling-related phenomenon also provides guidance for the required helium injection rate as the damage level increases. At 15–35 dpa (Fig. 7a) the ratio of helium generation rates at the maximum swelling is approximately a full order of magnitude. However, by 150 dpa (Fig. 7c), a transition from cavity nucleation to growth occurs, and this reduces the quantity of helium needed to stabilize cavity nuclei, and correspondingly the required helium injection rate is decreased. This decrease in helium injection needed for ion irradiations is also consistent with the time delay of helium partitioning to strongly bound cavities as described in Figs. 5 and Fig. 6. It is worth noting that all CD results presented incorporate a bias on small cavities which was necessary to reproduce bi-modal size distributions, incubation periods, and helium dependencies. However, it is important to note that the CD model with this mechanism tends to underpredict cavity density and overpredict cavity size. At high helium generation rates, this results in linear swelling rates orders of magnitude higher than experimental observation.

From these predictions, the dual ion irradiations, conducted at a damage rate 10^3^ × higher than that of the BOR-60 reactor, and with an increase in temperature (~ + 70 °C) and an increase in the He/dpa ratio (~ 18 ×), resulted in nearly identical cavity microstructures across the five conditions examined, as shown in Fig. 1. At low temperatures (376 °C in reactor and 445 °C with ions), both modes of the bimodal cavity distribution were reproduced. At high temperatures (460 °C in reactor and 520 °C with ions), no cavities larger than 3 nm radius were observed (Fig. 1d). This is consistent with the cavity growth rate calculations in Fig. 2b for reactor and Fig. 3b for ions where thermal emission is greater than net absorption at these cavity sizes and with the CD predictions (Fig. 4c, d) where swelling is not predicted. Thus, the capability has been established to predict the cavity microstructure in a nuclear reactor using ion irradiation in a laboratory accelerator.

The replication of the cavity microstructure with dual ion irradiation highlights the complex, interconnected nature of accelerated radiation damage experiments. This work indicates that enhanced cavity nucleation in reactor-associated helium trapping sites is necessary in the CD model to achieve the same void nucleation behavior as dual ion irradiation with homogeneous nucleation. For ion irradiations to reproduce reactor microstructures, they must be designed to capture the processes responsible for those microstructures. The combination of systematic experiments and mechanistic modeling of individual radiation damage processes has been shown to validate ion irradiation as a surrogate for neutron irradiation to predict structural material degradation over the lifetime of a component.

There are other processes that may contribute to the microstructures not considered in this analysis since the absolute amount of swelling predicted by the CD model is significantly higher than that of the experiment results. Helium accumulation in the strain fields of dislocations^30,31^ may reduce the interstitial bias of dislocations and reduce stress-induced heterogeneous nucleation. At high helium injection rates, such as those in the dual ion irradiation in this work, the buildup of helium and other impurities may shield the dislocation strain field, resulting in a reduced dislocation bias, perhaps to the level of a neutral sink. This effect would reduce the supersaturation of vacancies and lower cavity nucleation overall for homogeneous nucleation and delay the cavity growth for enhanced nucleation. This effect may be relevant at higher dpa where the continual supply of vacancies is needed for cavities to grow. Additionally, the effects of impurities in the microstructure^34^ will need to be considered to refine the calculated swelling values. Real microstructures may effectively achieve this enhanced cavity nucleation through microchemical effects (e.g., impurity segregation) or spatial correlations not captured in the models.

## Conclusions

The main conclusions from this work are as follows. First, higher damage rates in ion irradiation require higher irradiation temperatures to maintain a balance between defect production and loss; however, in this ferritic-martensitic alloy that also involves helium transmutation, the temperature shift is less than predicted by the Mansur invariance relationship. Second, higher rates of helium implantation are required in dual ion irradiations to compensate for the reduced ion irradiation time that impacts the distribution of helium among the microstructural features. As the irradiation continues, the impact of irradiation time becomes less as the distribution of helium shifts to stronger traps. In reactor, void nucleation behavior similar to dual ion irradiation with homogeneous nucleation can only be achieved by accounting for helium trapping and helium partitioning, which modify the temperature dependence of swelling by partially decoupling the cavity nucleation density from damage rate. The temperature dependence of swelling is governed by both the thermal evaporation of small vacancy clusters and helium trapping at damage-rate-independent trapping sites. These conclusions provide a guiding “formula” for predicting the complex radiation-induced phenomenon of cavity nucleation and growth at an accelerated damage rate. This achievement should pave the way for the discovery and design of new materials for advanced, carbon-free nuclear energy systems.

## Methods

This study was conducted on the ferritic-martensitic steel, T91 heat 30176. This same heat was used in previous studies on irradiation effects in the BOR-60 fast reactor^19^ and dual ion irradiation^20,21^ with the chemical composition received from an independent chemical analysis included in Table 2.

**Table 2:** Chemical compositions (wt%) of T91 heat 30176 provided by PNNL and Luvak; reproduced from Ref.^19^.

	Fe	Cr	Mo	C	N	Al	Si	P	S	Ti	V	Mn	Ni	Cu	Nb	W
wt%	Bal	8.76	.86	.091	.052	.004	.25	.007	.002	.003	.23	.44	.10	.062	.086	.004

The experiments in this work were conducted in separate irradiation campaigns described in Refs.^19–21^ and used as a comprehensive dataset for this work. T91 heat 30176 was irradiated with neutrons in the BOR-60 fast reactor at several temperatures in the range of 376–524 °C with an estimated uncertainty of ± 17 °C and to doses up to 35 ± 2 dpa with estimated damage rates between 6–9 × 10^−7^ dpa/s and a helium generation rate of 0.22 appm He/dpa calculated using the FISPACT-II multiphysics package^35^. Complementary dual ion irradiations were performed using the dual beam configuration at the Michigan Ion Beam Laboratory^36^ at damage rates of 5–8 × 10^–4^ dpa/s using 5 MeV iron ions with a helium co-injection rate of about 4 appm He/dpa to a total damage of up to 35 dpa at temperatures of 406–570 °C. Additional ion irradiations were conducted on T91 to isolate the role of irradiation damage rate and co-injected helium^21^. The damage rate series was performed with a helium co-injection rate of about 4 appm He/dpa to a total damage of 16.6 dpa at 445 °C with damage rates between 5 × 10^–5^ dpa/s and 3 × 10^–3^ dpa/s. The estimated uncertainty in the He/dpa is ± 10%, and the uncertainty in the dual ion irradiation damage rate is ± 8%. Because of the non-uniform damage profile produced using ion irradiation, characterization was limited to 500–700 nm from the surface. Additional details can be found in reference^21^ and in the supplemental materials for this manuscript.

The characterization of irradiated T91 from BOR-60^19^ and dual ion irradiation^20,21^ is summarized here. The same T91 samples from the BOR-60 reactor examined in Ref.^19^ were reexamined using TEM with a higher resolution camera to clarify the cavity size distribution in the 0–2 nm radius range. Cavities were characterized using high-angle annular dark field scanning transmission electron microscopy (HAADF-STEM). Additional characterization was performed to identify cavities smaller than 2 nm in radius using overfocused and underfocused bright field (BF) TEM imaging with a Gatan OneView 16-megapixel CCD camera capable of 4 K resolution with 0.25 nm point-to-point resolution. Hand-counting techniques were used with the FIJI image software^37^ to measure the cavity diameter to convert to a cavity radius and estimate the density of cavities from resulting images. Images for cavities can be found in the supplemental materials for this article. Dislocation loops were imaged using on-zone STEM BF imaging using a JEOL 2100F at the Michigan Center for Materials Characterization (MC^2^) near the [001] or [011] zone axis to view dislocation loops on edge, or nearly on edge, to distinguish between **a** < 100 > dislocation loops, **a**/2 < 111 > dislocation loops, and dislocation lines. Images for dislocation loops can be found in the supplemental materials for this paper. The foil thickness was measured in the STEM mode using the electron energy loss spectroscopy (EELS) zero loss method.

The cavity growth rate used in this work is expressed from Refs.^22,23^ as

5 $$\frac{dr}{{dt}} = \frac{\Omega }{r}\left[ {D_{v} C_{v} - D_{i} C_{i} - D_{v} C_{v,T} \exp \left( {\frac{2\gamma }{r} - p_{g} } \right)} \right].$$

The cavity growth rate equation is phenomenologically based on cavity growth from the absorption of vacancies (*D*_*v*_*C*_*v*_), shrinkage from absorption of interstitials (*D*_*i*_*C*_*i*_), and shrinkage from thermal emission of vacancies using the capillarity approximation with an offset from the pressure of gas (*p*_*g*_) in the cavity. Determination of the cavity growth rate involves two principle calculations: (1) the steady-state point defect concentrations and (2) the helium gas pressure inside a cavity. To calculate the point defect concentrations, a standard rate equation for the change in defect concentration of either interstitials and vacancies with time was used from Ref.^38^:

6 $$\frac{{dC_{{\left( {i,v} \right)}} }}{dt} = G_{0} - R_{iv} C_{i} \left( {C_{v} + C_{v,T} } \right) - k_{{\left( {i,v} \right)}}^{2} D_{{\left( {i,v} \right)}} C_{(i,v)} ,$$

where $k_{{\left( {i,v} \right)}}^{2}$ is the sink strength for either vacancies or interstitials and $D_{{\left( {i,v} \right)}}$ is the diffusion coefficient for the point defect specie. The first term on the right side of the equation is the production rate of defects, $G_{0}$. The second term, $R_{iv} C_{i} \left( {C_{v} + C_{v,T} } \right)$, is the annihilation of the point defects due to mutual recombination and includes the loss of interstitials recombining with thermally produced vacancies. The final term in the equation is the loss of point defects to sinks.

The recombination parameter for defects was taken from Ref.^38^:

7 $$R_{iv} = \frac{{z_{iv} \Omega \left( {D_{i} + D_{v} } \right)}}{{a^{2} }},\quad z_{iv} \approx 500,$$

where $z_{iv}$ is the combinatorial factor, ${\Omega }$ is the atomic volume, and $a$ is the lattice parameter.

The sink strengths for interstitials and vacancies were calculated for each irradiation condition using the microstructure characterized and presented in the results. For each specie, the total sink strength is the sum of the sink strengths of the individual measured sinks:

8 $$k_{v}^{2} = k_{dis}^{2} + k_{cav}^{2} + k_{gb}^{2} ,$$

9 $$k_{i}^{2} = k_{dis}^{2} \left( {1 + Z} \right) + k_{cav}^{2} + k_{gb}^{2} ,$$

where $k_{cav}^{2}$ is the sink strength from cavities and bubbles, $k_{gb}^{2}$ is the grain boundary sink strength, and the dislocation sink strength, $k_{dis}^{2}$, is multiplied by an interstitial bias factor, *Z*. The sink strengths of precipitates were found to be negligible compared with the dislocation loops and cavities, as discussed in the supplemental materials.

The diffusion of interstitials and vacancies was assumed to have an Arrhenius dependence:

10 $$D_{i,v} = \alpha_{i,v} \omega_{i,v} \exp \left( { - \frac{{E_{m}^{i,v} }}{kT}} \right),$$

where $\alpha$ is 1/6 for interstitials and 1 for vacancies in body centered cubic lattices, $\omega$ is the jump frequency (s^−1^) for either vacancies or interstitials, $k$ is the Boltzmann constant, $T$ is the temperature in Kelvin, and $E_{m}$ is the migration energy for the point defect specie (eV).

Finally, the thermal vacancy concentration ($C_{v,T}$) was estimated using

11 $$C_{v,T} = \frac{1}{\Omega }\exp \left( {\frac{{S_{f} }}{k}} \right)\exp \left( { - \frac{{E_{f}^{v} }}{kT}} \right),$$

where $S_{f}$ is the entropy of formation (eV/K), and $E_{f}^{v}$ is the vacancy formation energy (eV). The thermal interstitial concentration was assumed to be negligible.

All of the previously described equations were used with the time derivative in Eq. (2) set to zero to solve for the steady state concentration of interstitials and vacancies using a numerical solver in MATLAB®. The pressure of helium gas inside a cavity was calculated using a modified form of the ideal gas law to include a hard sphere equation of state for helium, as used in previous work on cavity nucleation^22,39^:

12 $$p_{g} = \frac{{n_{g} k_{b} TZ_{comp} }}{V},$$

where $V$ is the volume of the spherical cavity, $n_{g}$ is the number of gas atoms with a factor for the compressibility of the helium gas,

13 $$Z_{comp} = \frac{{1 + y + y^{2} + y^{3} }}{{(1 - y)^{3} }},$$

where

14 $$y = \pi n_{g} \frac{{d_{g}^{3} }}{{6V^{3} }},$$

and

15 $$d_{g} = 0.3135\left( {0.8542 - 0.03996\ln \left( {\frac{T}{9.16}} \right)} \right),$$

where $d_{g}$ is the hard sphere diameter of helium.

These equations were used to solve for the pressure of the gas numerically using MATLAB. The default parameters used for the calculation of the cavity growth rate equation are included in Table 3. The dislocation bias for interstitials has been reported in literature in the range of 1 to 25% using analytic solutions^40–42^ and 1 to 5% using rate theory approaches^23,43–45^. A value of 5% was chosen for the cavity growth rate analysis to include the largest effect in the range of both approaches. Similarly, a range of vacancy migration energies from 0.57 to 0.69 eV^46–49^ was found in literature, and a value of 0.62 eV was chosen for the cavity growth rate to be near the middle of the range. The uncertainty for the calculations was done using standard error propagation techniques^50^ from the uncertainty in the input parameters in Table 3.

**Table 3:** Table of input parameters for calculating the cavity growth rate equation and CD when different from the cavity growth rate input parameters.

Parameter	Value for cavity growth rate	Value for CD	References
Temperature, T (°C)	Input parameter	Input parameter	NA
Damage Rate, K_0_ (dpa/s)	Input parameter	Input parameter	NA
Helium Co-Injection Rate (appm He/dpa)	Input parameter	Input parameter	NA
N	8.34 × 10^22^ at/cm^3^	Same	^46^
Lattice parameter (a)	0.288 nm	Same	^46^
Sink strength	From microstructure	From cluster evolution	This work
ω_i_	2.9 × 10^12^ s^-1^	Same	^46^
ω_v_	1.6 × 10^13^ s^-1^	Same	^46^
γ	1.75 J/m^2^	Same	^55^
$E_{m}^{v}$	0.62 eV	0.63 eV	See text
$E_{f}^{v}$	1.6 eV	1.79 eV	^46^
Di-vacancy binding energy	N/A	0.3 eV	^56^
$E_{m}^{i}$	0.35 eV	0.22 eV	^46^
S_f_	2.17 k	Not used	^38^
Dislocation Bias, Z	5%	15%	See text
E	220 GPa	Not used	^57^
ν	~ 0.3	Not used	^58^

A model of helium trapping and de-trapping was constructed^21^ from a mass balance approach as a set of differential equations. It consists of the generation or injection of helium, the trapping of helium at a feature in proportion to the sink strength, and the thermal de-trapping of helium from a feature:

16 $$\frac{{dC_{HeV} }}{dt} = G_{He} - \mathop \sum \limits_{i}^{{\begin{array}{*{20}c} {\text{loops, bubbles,}} \\ {\text{HeV clusters, lines}} \\ \end{array} }} k_{i}^{2} D_{He} C_{HeV} + \mathop \sum \limits_{i}^{{\begin{array}{*{20}c} {\text{loops, bubbles,}} \\ {\text{HeV clusters, lines}} \\ \end{array} }} N_{i} \Omega n_{g}^{i} \nu_{0} \exp \left( { - \frac{{E_{b}^{i} }}{kT}} \right),$$

where $C_{HeV}$ is the concentration of helium in the matrix, $G_{He}$ is the helium injection rate, $k_{i}^{2}$ is the sink strength microstructure feature *i,* such as dislocation loops, bubbles, and dislocation lines, $N_{i}$ is the density of a group of microstructure feature *i*, $k$ is the Boltzmann constant, $T$ is the irradiation temperature, Ω is the atomic volume, and $E_{b}^{i}$ is the binding energy of helium to microstructure feature *i*. Binding energies for helium attachment used were 1.0 eV for dislocation lines^30^, 2.3 eV for dislocation loops^31^, 2.3 eV for HeV clusters^32^, and 3.3 eV for cavities^33^. For dislocations, the strongest binding energy in the tension field of the dislocation was used. The remaining term in the equation is the number of helium atoms per microstructure feature, $n_{g}^{i}$, which can be described as a separate mass balance rate equation for each feature using

17 $$\frac{{dn_{g}^{i} }}{dt} = \frac{{k_{i}^{2} D_{HeV} C_{HeV} }}{{N_{i} \Omega }} - n_{g}^{i} \nu_{0} \exp \left( { - \frac{{E_{b}^{i} }}{kT}} \right).$$

The details and solution methods for the equations to determine the point defect concentrations and material parameters that go into these equations are described in Ref.^21^.

The microstructure contains other sinks for helium and point defects, such as lath boundaries, packet boundaries, prior austenite grain boundaries, coarse M_23_C_6_ carbides, fine V, Cr-nitrides, and Ni/Si clusters that serve as precursors for G-phase precipitates^19,21^. The grain boundary sink strength was smaller than the sink strengths of cavities and dislocation loops in most cases and always lower than the initial dislocation line sink strength of about 5 × 10^14^ m^-2^ (Ref.^21^). Compared with the other sinks, the sink strength of grain boundaries is at most 7% of the total sink strength. The preexisting precipitates and radiation-induced clusters have a negligible sink strength compared with dislocation loops and cavities^21^, and therefore they were neglected from the total sink strength calculation.

Several assumptions about the early interactions of helium and point defects were included in the helium trap and release approach. First, it is assumed that all helium atoms that enter the lattice quickly become substitutional helium atoms (HeV clusters). In reactor, helium in structural materials is generated as an energetic alpha particle (He^2+^) from (n,α) reactions. These helium ions, whether from reactor or co-injected during ion irradiation, will generate energetic primary knock-on atoms (PKAs) that can cause displacement cascades and produce vacancies. As the helium ions come to rest, they are unlikely to encounter a vacancy created by the helium-induced PKAs^51^. However, the vacancies produced from neutrons and ions greatly outnumber the helium-produced defects. Because interstitial helium has a high thermal diffusivity ($D_{0}^{He}$ ~ 2.8 × 10^–8^ m^2^s^-1^,$E_{m}^{He}$ ~ 0.064 eV from Ref.^52^), it is expected to quickly encounter and bind to a vacancy. Thus, all helium in the lattice is assumed to diffuse as substitutional helium with an Arrhenius dependence from a pre-factor of $D_{0}^{HeV}$ ~ 6.4 × 10^–7^ m^2^s^-1^ and a migration energy of $E_{m}^{HeV}$ ~ 0.32 eV from Ref.^53^. Second, as these clusters cannot be readily identified with TEM, a conservative assumption was made that the density of HeV clusters is equal to the steady state concentrations of radiation-induced vacancies, $C_{v}$. The HeV clusters were assumed to behave like small cavities with regard to helium and have the same formulation for calculating their sink strength.

The CD model, based on the SPICES code^27^, employs an intricate network of interconnected differential equations for each allowed cluster size. For a given species *j*, the concentration is governed by

18 $$\frac{{dC_{j} }}{dt} = G_{j} + R_{j} \left( C \right) - \mathop \sum \limits_{{m = 1,n_{{{\text{sinks}}}} }} k_{{{\text{sink}}}}^{m} D_{j} C_{j} ,$$

where $R_{j} \left( {C_{j} } \right)$ is a sum of all possible aggregation events,

19 $$R_{j}^{ + } \left( C \right) = \mathop \sum \limits_{n + m \to j} k_{n,m}^{ + } C_{n} C_{m} - \mathop \sum \limits_{k} k_{j,k}^{ + } C_{j} C_{k} ,$$

and dissociation events,

20 $$R_{j}^{ - } \left( C \right) = \mathop \sum \limits_{n \to n - j,j} k_{n - j,j}^{ - } C_{n} - \mathop \sum \limits_{k} k_{j,k}^{ - } C_{j} ,$$

which create or consume a species *j*, where *k*’s are unique rate constants encapsulating the kinetics of each reaction. When expanding the steady state calculations to a CD approach, several additional mechanisms and treatments were included. Decades of simulations show high-energy damage cascades generate larger clusters than used in the steady state model, and thus, the CD model includes primary damage that generates up to nine-member vacancy clusters. The nucleation and growth of a visible cavity microstructure is the result of a series of individual defect capture events by smaller clusters through aggregation and shrink through dissociation. These reactions are described in more detail in the supplemental materials.

An enhanced nucleation mechanism was incorporated through the addition of a parallel phase space associated with a preferred nucleation site. A constant density of immobile nucleation sites was introduced to the system, which immobilized helium atoms according to a trapping/de-trapping pair defined in Eqs. (19) and (20), where de-trapping rates (*k*^*−*^) are governed by an input binding energy, providing a static site for self-clustering in the absence of vacancies. Eventually these helium clusters will achieve sufficient pressure to punch out an interstitial, or absorb a vacancy, transforming into a bubble. The aggregation-emission behavior of larger helium clusters is presumed to have the same clustering energetics as free interstitial helium in the lattice according to Gao^54^, with only mobility affected.

## Supplementary Information

Supplementary Information.
