# Supplementary material for: Predicting structural material degradation in advanced nuclear reactors with ion irradiation
Source: Sci Rep. 2021 Feb 3;11:2949. doi: 10.1038/s41598-021-82512-w (PMC7859217; doi:10.1038/s41598-021-82512-w)
Supplement: Supplementary file 1 — Supplementary Information. [file 41598_2021_82512_MOESM1_ESM.docx]

**Supplemental Materials to “Predicting Structural Material Degradation in Advanced Nuclear Reactors with Ion Irradiation”**

Stephen Taller^[[1]](#footnote-2)^*^1,2^, Gerrit VanCoevering^1^, Brian D. Wirth^3^, Gary S. Was^1^

1-Department of Nuclear Engineering and Radiological Sciences, University of Michigan, Ann Arbor, MI, US

2-Currently at Nuclear Energy and Fuel Cycle Division, Oak Ridge National Laboratory, Oak Ridge, TN, US

3-Nuclear Engineering, University of Tennessee–Knoxville, Knoxville, TN, US

1. **Comparison of the Primary Damage from Reactor and Ion Irradiation**

The interactions between energetic particles and a solid crystal are complex and result in primary damage from collision events ^1^. These energetic particles, whether neutrons, ions, or electrons, produce primary knock-on (or recoil) atoms (PKAs) when enough energy is transferred to an atom in the crystal lattice. These recoil atoms can create subsequent collision events, leading to cascading damage events. A full description of the theory behind damage accumulation from radiation damage from these damage cascades is beyond the scope of this work and available in Ref. ^1^. When comparing reactor and ion irradiation, some care must be taken to replicate this primary damage reasonably well.

For defect production, the absolute number of recoils of a specific energy are not as important as the number of recoils in each collision event to be transferred to subsequent cascades ^2^. To compare the primary damage between reactor and ion irradiation, a weighted recoil spectra was calculated for the neutron flux–energy spectra for the BOR-60 reactor using the program SPECTER ^3^ and 5 MeV iron ions in the range of interest 500–700 nm from the surface using the Stopping and Range of Ions in Matter (SRIM) program ^4^ to guide the experimental parameters and characterization. The weighted average recoil spectra are shown in Figure A.1. The weighting factor is a simple scaling factor to convert the primary recoil spectra by the number of defects produced according to a hard sphere potential for neutron collisions and a Lindhard screened Coulomb potential for iron ions to account for electronic excitations.


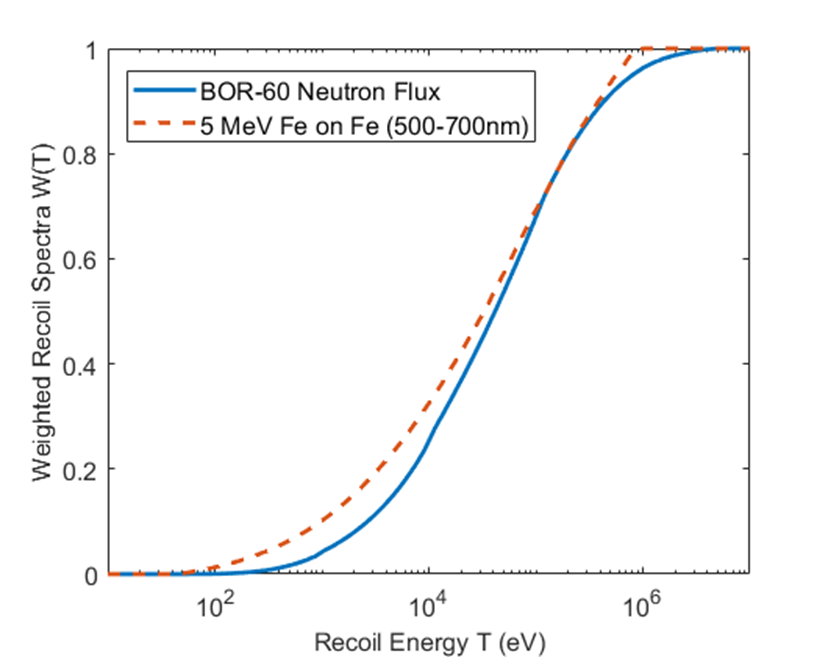


Figure A.1. Comparison of the weighted average recoil spectra for the BOR-60 fast reactor and energetic 5 MeV iron ions in pure iron.

The weighted recoil spectra exhibit the same shape between the two conditions, with small deviations in the tails of the distribution at high and low energy. Decades of molecular dynamics simulations of high PKA energy damage events have demonstrated the propensity to form defect clusters directly in cascades. This can be folded into the PKA distributions to generate a vector of defect cluster production terms for the cluster dynamics (CD) calculations using a procedure outlined by Xu and coworkers ^5^. One such vector was created (Figure A.2), which generates clusters as large as nine vacancies, in accordance with molecular dynamics (MD) simulations conducted in pure iron.


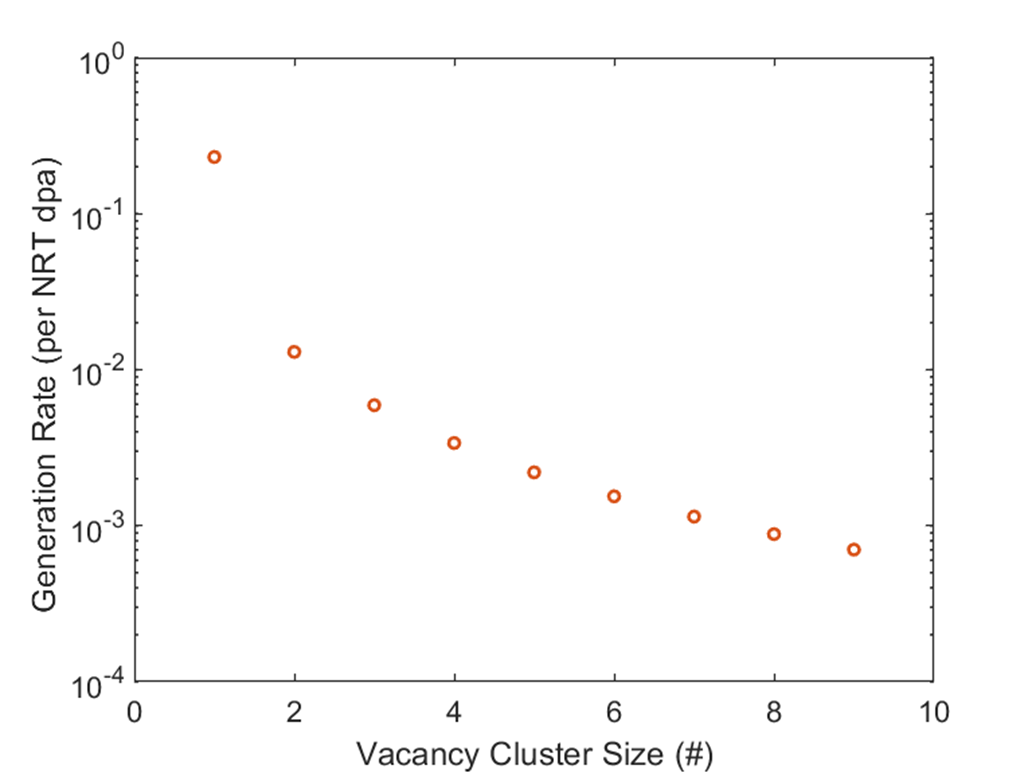


Figure A.2. Vacancy cluster distributions used in cascade primary damage formulation.

1. **Characterization of the Microstructure of Alloy T91**

As stated in the main paper, the experiments in this work were conducted in separate irradiation campaigns as described in Refs. ^6–8^ and used as a comprehensive dataset for this work. Characterization from these references is included to provide parameters and comparisons for the rate theory and CD models. Due to the shallow penetration depth of the Fe^2+^ and He^2+^ ions in the dual ion–irradiated T91 and the residual radioactivity of the BOR-60–irradiated T91, the FIB (focused ion beam) lift-out method was needed to prepare TEM samples. This method allowed for the extraction of cross-sectional slices of material just a few microns into the surface. Additionally, T91 is highly magnetic, and large samples would cause magnetic distortion in the electron beam during TEM imaging. The FIB lift-out method allowed for microstructural analysis and avoided magnetic issues during imaging. The TEM foils were prepared using a standard dual-beam FIB process. A low-energy ion beam (5 keV and 2 keV) was used when the foil was still relatively thick (around 150 nm) to thin the foil to a final thickness of around 100 nm, which effectively eliminated TEM-visible FIB damages induced at high beam energy. The specific instruments used in this work were the ThermoFisher Scientific (TFS) Helios NanoLab™ Dual Beam™ and Nova NanoLab™ DualBeam™ FIB workstations for the dual ion–irradiated T91 at the Michigan Center for Materials Characterization (MC^2^) and TFS Versa NanoLab™ Dual Beam™ and Quanta NanoLab™ DualBeam™ for the BOR-60-irradiated T91 at the Low Activation Materials Development Analysis (LAMDA) laboratory ^9^ at Oak Ridge National Laboratory (ORNL).

Consistent procedures were used to quantify the radiation-induced defects in BOR-60 and dual ion–irradiated T91. Dislocation loops and cavities were characterized using a JEOL 2100F TEM/STEM at either the LAMDA laboratory or MC^2^. The STEM-BF (bright field) technique was used for dislocation imaging as it resulted in reduced strain contrast compared with the traditional TEM two-beam BF technique ^10,11^. As the majority of dislocation loops in irradiated ferritic-martensitic steels are of the type a<100>, they were characterized using either the <001> or <011> zone axis, under which a fraction (2/3 under <001> zone axis and 1/3 under <011> zone axis) of a<100> loops appears edge-on in the image. Cavities were characterized using high-angle annular dark-field scanning transmission electron microscopy (HAADF-STEM). Additional characterization was performed on the JEOL 2010F at MC^2^ to identify cavities smaller than 2 nm in diameter using overfocused and underfocused BF TEM imaging with a Gatan OneView 16-megapixel CCD camera capable of 4k resolution with 0.25 nm point-to-point resolution. Hand-counting techniques were used with the FIJI image software ^12^ to measure the cavity diameter and estimate the density. The swelling was calculated from measured cavities using the following equation:

$\frac{\Delta V}{V}=\frac{\frac{4\pi}{3}\sum_{i=1}^{N} r_{i}^{2}}{A\delta-\frac{4\pi}{3}\sum_{i=1}^{N} r_{i}^{2}}\times100\%$ ,

(A.2)

where *A* is the area of the images, *δ* is the foil thickness, *r_i_* is the cavity diameter, and *N* is the number of cavities. The foil thickness was measured in the STEM mode using the electron energy loss spectroscopy (EELS) zero loss method. X-ray count maps were obtained for precipitate characterization using the FEI Talos F200X STEM instrument at LAMDA equipped with high counting rate energy dispersive x-ray spectroscopy (EDS). EDS-based spectrum images were taken using a region-of-interest size of 1024×1024 pixels with a resolution of ~0.23 nm/pixels with a probe full width half max of ~1.5 nm and a beam current around 3 nA. Each scan had a duration of 1 hour with more than 35,000 counts/sec with dead times from 1–6%.

- 1. As-received Characterization

The unirradiated microstructure of T91 in the as-tempered condition consisted of prior austenite grain boundaries, packets, laths, coarse chromium carbides, fine V,Cr-nitrides, and network dislocations (Figure A.3). Chromium carbides were mainly M_23_C_6_ type and were mostly on the prior austenite grain boundaries (PAGBs) or lath boundaries. Fine V,Cr-nitrides were observed in the matrix and sometimes were found adjacent to the Cr-carbides.


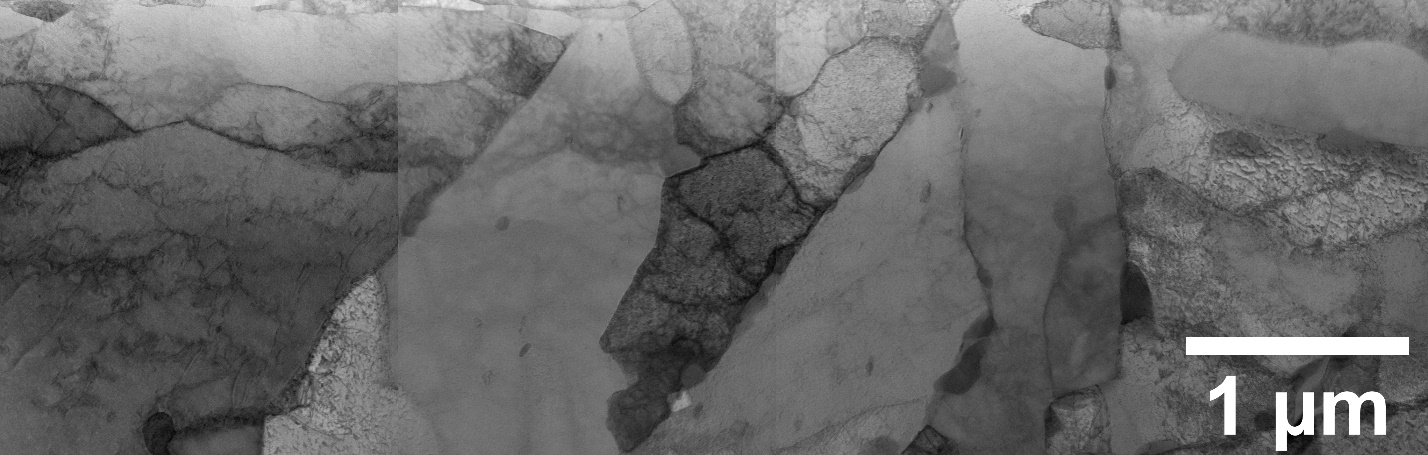


Figure A.3. Bright field scanning transmission electron microscopy image of T91 heat 30176 in the as-received, as-tempered state. Reproduced from Ref. ^7^ with permission.

The size of preexisting precipitates is described an equivalent diameter, given as

$d_{eq}=\sqrt{a\times b}$,

(A.1)

where *a* and *b* are the lengths of the major and minor axis of the precipitate, respectively. The average size of M_23_C_6_ was estimated to be ~100 nm, while the average size for (V,Cr)N was approximately 40 nm. Both Cr-carbides and V,Cr-nitrides had an estimated number density between 10^18^ m^-3^ and 10^19^ m^-3^ based on the EDS-based spectrum images and foil thickness measurements. The dislocation density varied from area to area and was estimated to be ~10^15^ m^‑2^. The average grain size was calculated using Eq. (A.1) measuring the grain width at its widest and narrowest points from STEM BF images to be about 800±50 nm.

- 1. BOR-60 Irradiation

BOR-60-irradiated T91 samples were received and characterized at the LAMDA laboratory at ORNL ^9^.

- - 1. Cavities

In addition to the STEM HAADF images presented in the main article, conventional transmission electron microscopy (CTEM) BF underfocused images (Figure A.4) showed cavities at sizes smaller than those observed with STEM. These images correspond to the irradiation conditions described in Figure 1 in the main article. Small cavities were observed in all BOR-60 irradiation conditions and largely contributed to the first peak in the cavity size distributions shown in Figure 1 in the main article.


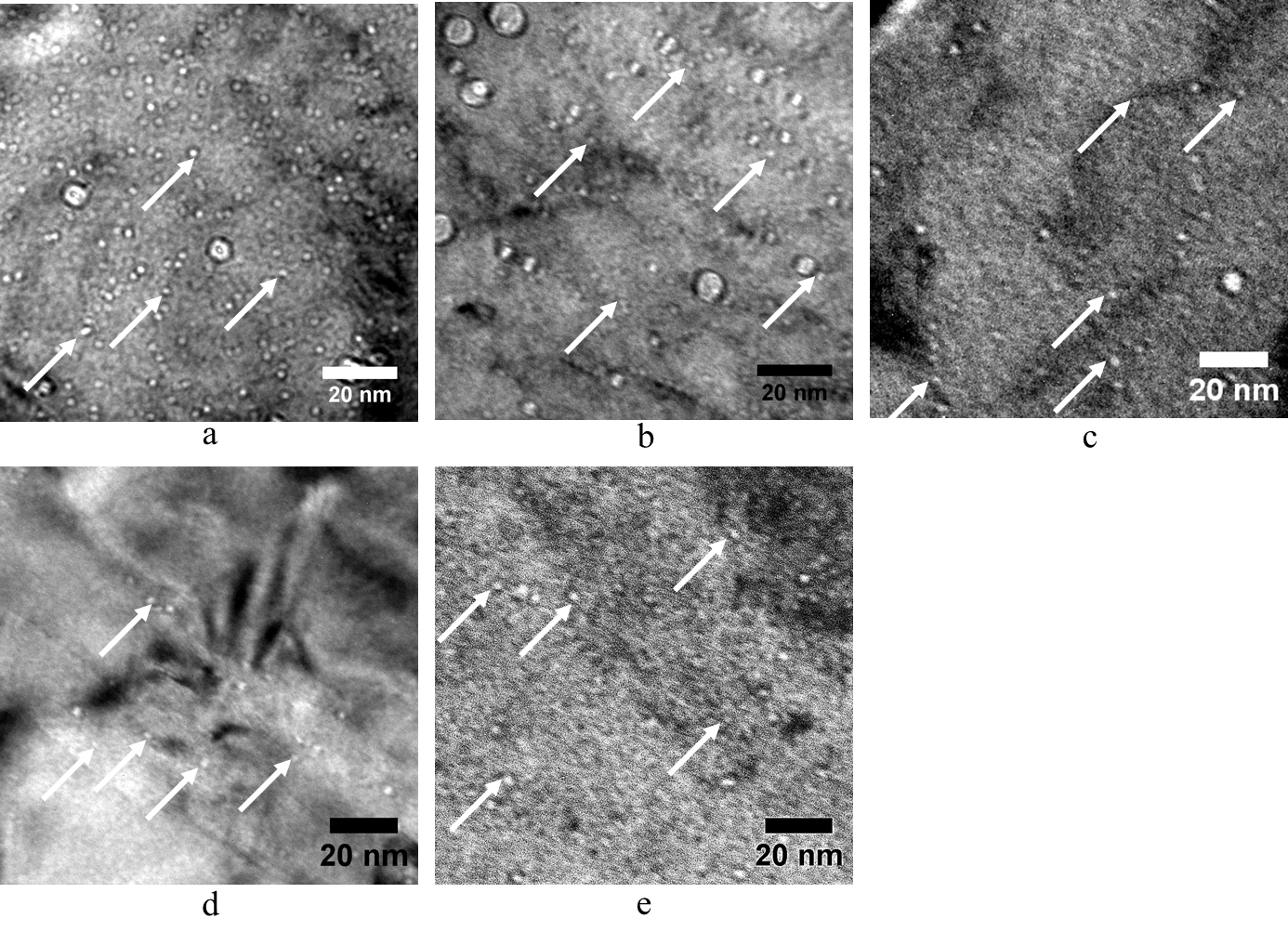


Figure A.4. CTEM BF underfocused micrographs at about -1 µm focus of T91 specimen irradiated in the BOR-60 reactor corresponding to the conditions of Figure 1 in the main article: (a) 376°C, 17 dpa, (b) 378°C, 35 dpa, (c) 415°C, 17 dpa, (d) 460°C, 15 dpa, and (e) 524°C, 15 dpa.

- - 1. Dislocation Loops

As stated previously, micrographs for dislocation loops were captured using STEM BF imaging. Representative micrographs for the BOR-60 irradiation conditions described in the main article are included as Figure A.5, with the resulting size distributions in Figure A.6. All irradiation conditions show the dominance of **a**<100> loops compared with loops with Burgers vector **a**/2<111>. There was a slight increase in average loop diameter and decrease in number density at 35 dpa compared with 17 dpa due to the absence of smaller loops (< 10 nm) in this condition, although the number density appeared to drop in nearly all the loop size bins. As the temperature increased, the density of dislocation loops decreased until none were observed at 460°C or 524°C. The size distributions were used to calculate the sink strength of the dislocation loops, as detailed in a later section.


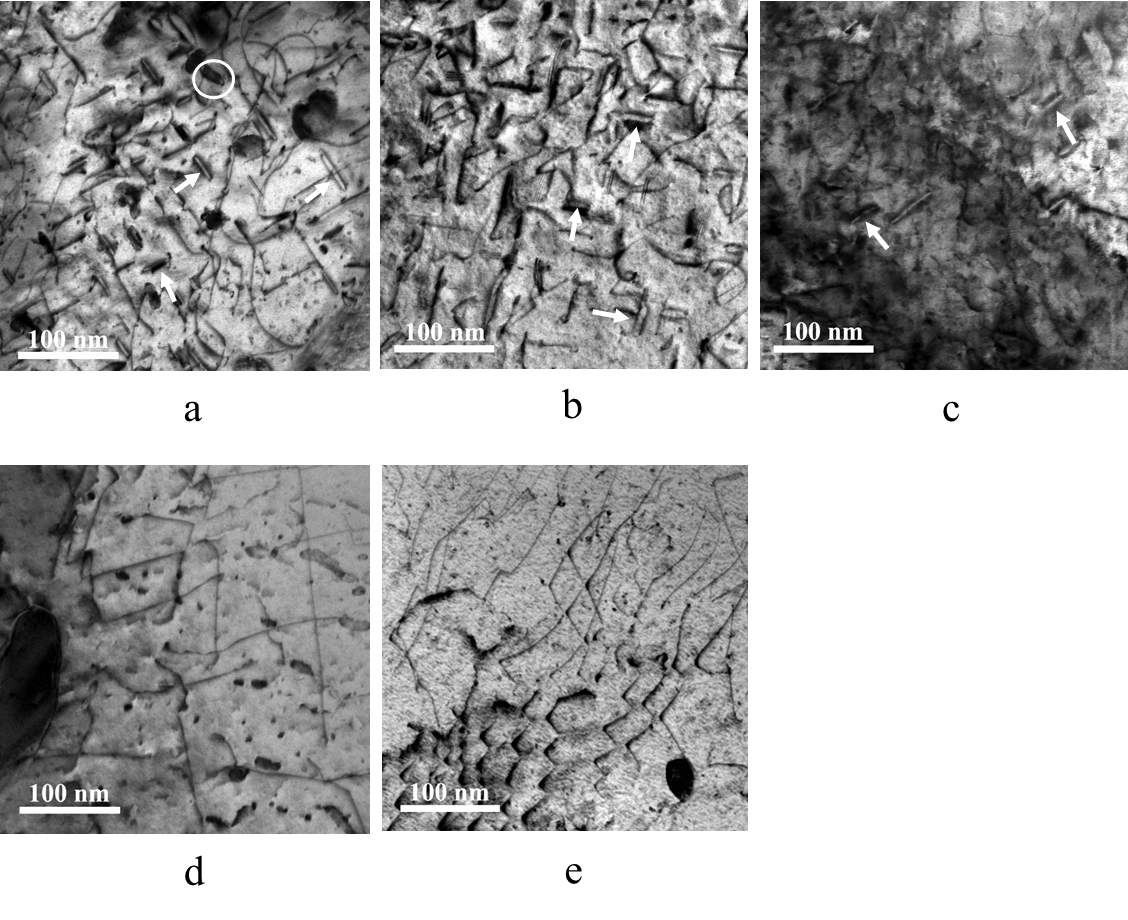


Figure A.5. STEM-BF images showing dislocation evolution in T91 irradiated in BOR-60 under different irradiation conditions: (a) 17 dpa at 376°C; (b) 35 dpa at 378°C; (c) 17 dpa at 415°C; (d) 15 dpa at 460°C; and (e) 15 dpa at 524°C. Some near edge-on dislocation loops are indicated by arrows. A possible 1/2**a**<111> type dislocation loop is circled in (a). Reproduced from Ref. ^6^ with permission.


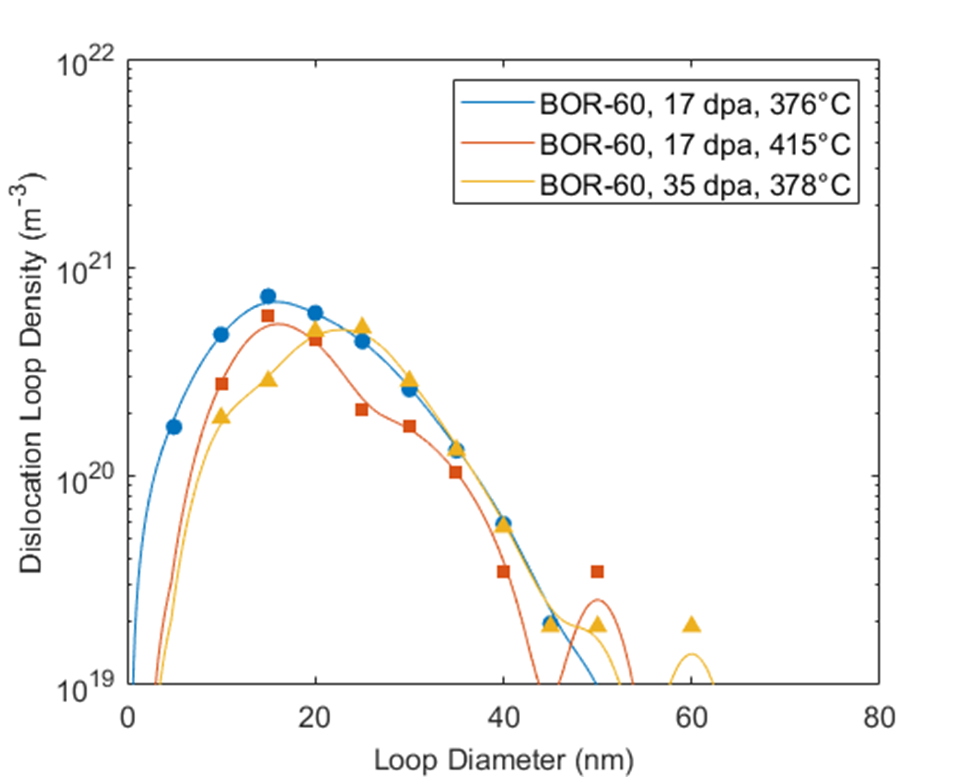


Figure A.6. Dislocation loop size distributions for BOR-60 irradiated T91.

- - 1. Ni/Si Clusters

The major radiation-induced precipitates were the Ni/Si-rich precipitates which can be observed in EDS maps produced from the Ni K-α x-ray signal in Figure A.7 for each of the BOR-60 irradiation conditions. The distribution of Ni/Si-rich precipitates is shown in Figure A.7f. The average precipitate size increased slightly from 17 dpa to 35 dpa at 376–378°C while the number density slightly decreased. Irradiation at 415°C resulted in slightly larger precipitates with lower density compared with the 376°C irradiation.


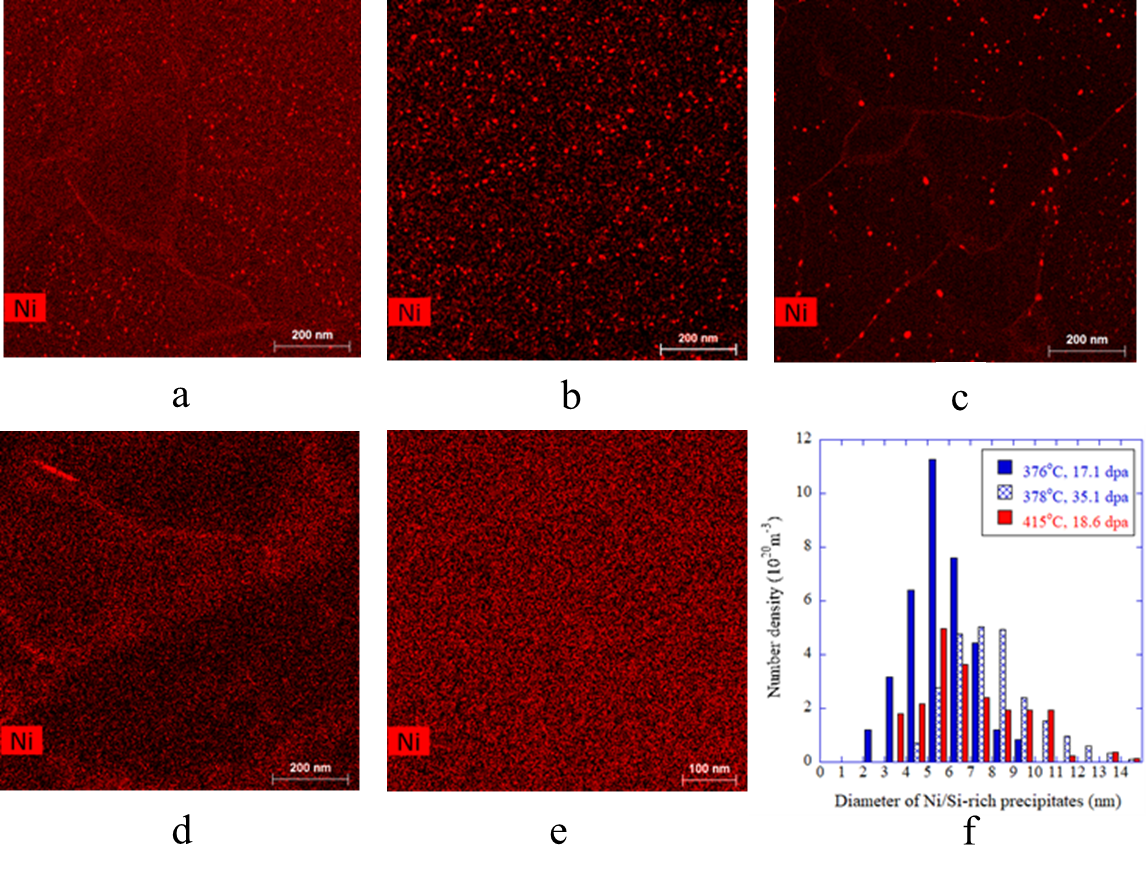


Figure A.7. Ni/Si-rich clusters evolution in T91 irradiated in BOR-60 under different irradiation conditions: (a) 17 dpa at 376°C; (b) 35 dpa at 378°C; (c) 17 dpa at 415°C; (d) 15 dpa at 460°C; (e) 15 dpa at 524°C; and (f) Ni/Si cluster size distributions. Reproduced from Ref. ^6^ with permission.

- 1. Dual Ion Irradiation

In these dual ion–irradiated samples, there is a non-constant damage profile from the 5 MeV iron ions. Following the considerations presented in literature for the effects of injected interstitials and the free surface, the valid area for analysis is 300–700 nm from the surface ^13,14^. Therefore, the region of analysis for all microstructural features considered in this work is 500–700 nm to limit the variation in damage and helium concentration over the examination area. For each irradiation condition, at least two FIB lamellas were produced from distinct areas on the irradiated surface. The cross-sectional area for the region of interest is at least 4.0 µm^2^ per irradiation condition to average over the inherent heterogeneity of the ferritic-martensitic microstructure.

- - 1. Cavities

Cavities were observed at all temperature-damage conditions, shown with STEM HAADF in Figure 1 in the main article and with CTEM BF in Figure A.8 to highlight the small cavities that form a large density in the size distributions. Although some large cavities were observed at 520°C, there were too few to obtain a reasonable average for the diameter or enough to make a statistically sound size distribution. The bubble number density exhibited no obvious trend with temperature at 15–17 dpa. An increase in dose from 17 dpa to 35 dpa at 445°C resulted in a modest increase of the average cavity diameter and almost doubling of the void density. The bubble density remained fairly constant with dpa.


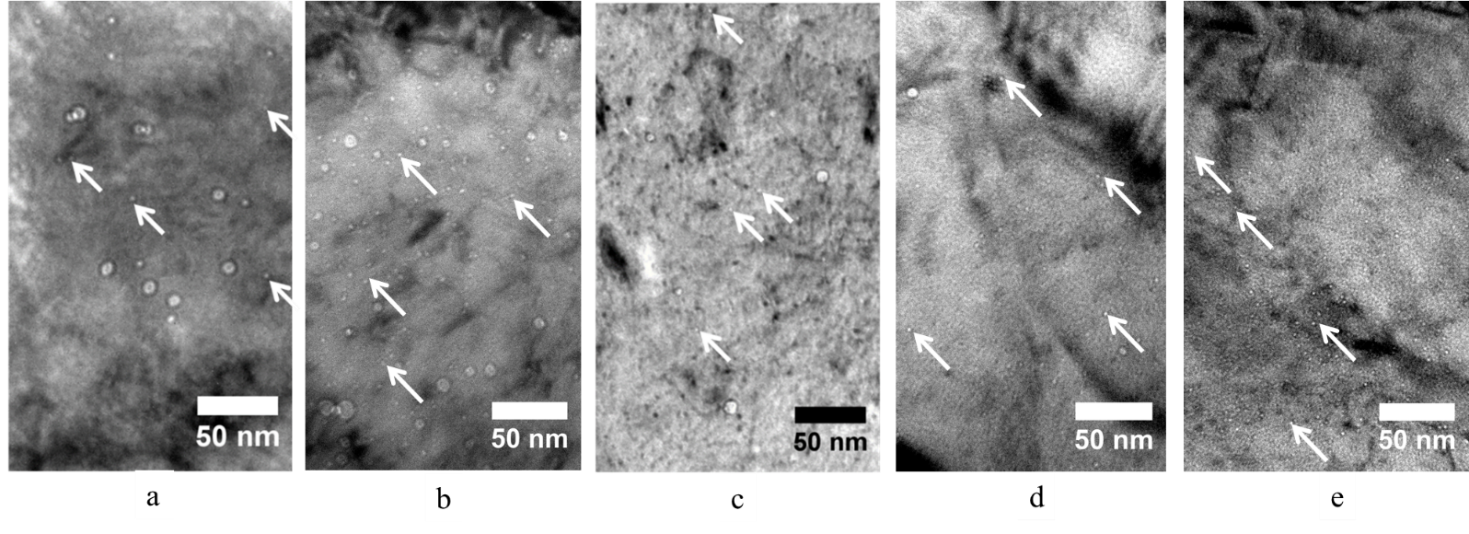


Figure A.8. CTEM-BF underfocused images showing cavitied in dual ion–irradiated T91 with 4 appm He/dpa under different irradiation conditions: (a) 17 dpa at 445°C, (b) 35 dpa at 445°C, (c) 17 dpa at 460°C, (d) 17 dpa at 520°C, and (e) 17 dpa at 570°C. Several small cavities are highlighted with arrows.

- - 1. Dislocation Loops

Loops with a Burgers vector of **a**<100> on either (010) or (001) planes appeared as nearly edge-on in the STEM BF images, as shown in Figure A.9, while the **a**/2<111> loops residing on (111) planes appeared at an angle of approximately 54° relative to the (001) plane. The primary dislocation loops observed at each temperature were **a**{100} dislocation loops. Very few **a**/2<111> dislocation loops were observed across all temperatures examined and comprised less than 10% of the total loop number density. At the higher temperature of 520°C:14.6dpa, very few dislocation loops were observed and there were not enough to have statistical significance. At the highest temperature of 570°C:15.4dpa, only dislocation lines were observed. Dislocation lines were observed at all temperatures, and no significant alteration of the existing network dislocation density was noted compared with the as-tempered condition. The resulting dislocation loop size distributions for calculation of the sink strength are displayed in Figure A.10.


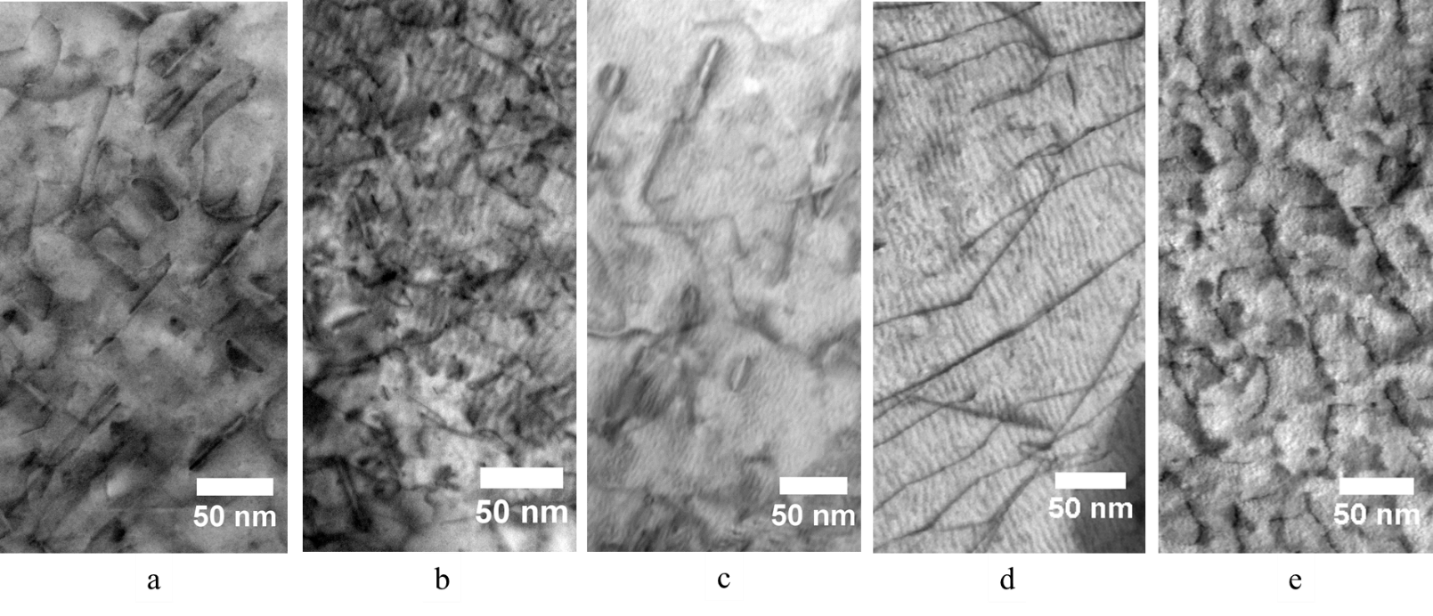


Figure A.9. STEM-BF images showing dislocation evolution in dual ion irradiated T91 with 4 appm He/dpa under different irradiation conditions: (a) 17 dpa at 445°C, (b) 35 dpa at 445°C, (c) 17 dpa at 460°C, (d) 17 dpa at 520°C, and (e) 17 dpa at 570°C. Reproduced in part from Ref. ^7^ with permission.


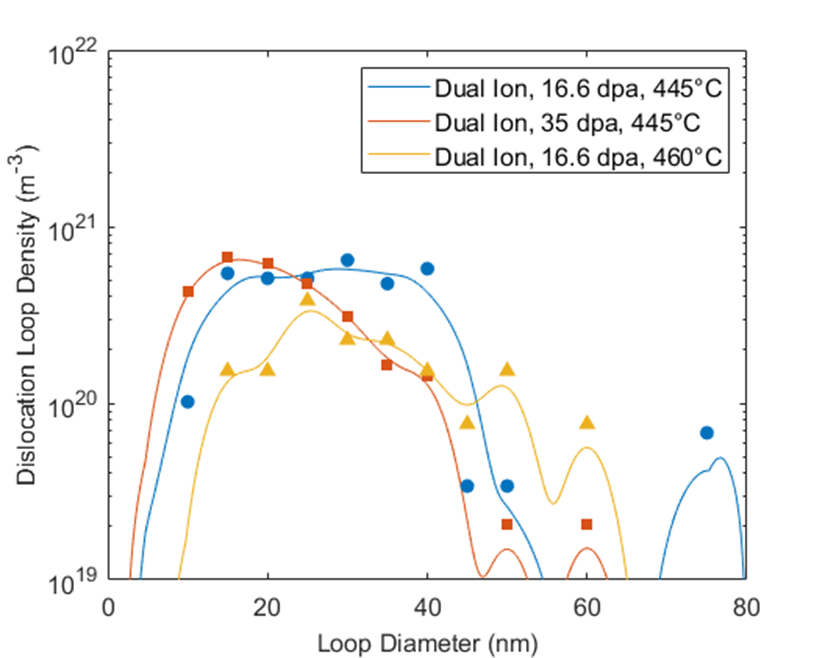


Figure A.10. Dislocation loop size distributions for dual ion irradiated T91.

- 1. Calculation of Sink Strengths for the Cavity Growth Rate Equation

The calculation of sink strengths from the characterization of the irradiated microstructure is based on the formula presented in Ref. ^2^ and discussed in the supplemental materials for Ref ^8^. The sink strength of the total cavity size distribution comes from integrating the size distribution, from Ref. ^2^:

$k_{cav}^{2}=4\pi\sum_{i=0}^{R} r_{i}N_{i}$,

(A.3)

where *N_i_* is the density of cavities of radius *r_i_*, and *R* is the maximum cavity radius observed. From the values of diameter and density in the dislocation loop size distributions, a total loop line length (or sink strength) was calculated. The sink strength of the dislocation loops was calculated from the size distribution:

$k_{loop}^{2}=\pi\sum_{i=0}^{D} d_{i}\rho_{i}$,

(A.4)

where *ρ_i_* is the number density of dislocation loops of diameter *d_i_* in the dislocation loop size distribution and $D$ is the maximum dislocation loop diameter observed. As discussed in Refs. ^15,16^, a higher boundary misorientation angle leads to a higher sink strength and thus the different types of boundaries in ferritic-martensitic steels will lead to differences in sink strength. Conservative estimate boundaries were treated as prior austenite grain boundaries (high sink strength) using the equation for the strongest sink strength boundaries with small grain diameters from Ref. ^2^ with an estimated grain diameter of 800 nm:

$k_{gb}^{2}=\frac{24}{d^{2}}$ . (A.5)

The resulting sink strength is about 3.7 × 10^13^ m^-2^. This sink strength was assumed to be constant for all irradiation conditions. This is lower than the sink strengths of cavities and dislocation loops in most cases (see Table 1 in the main manuscript) and always lower than the initial dislocation line sink strength of about 5 × 10^14^ m^-2^. Compared with the other sinks, the sink strength of grain boundaries is at most 7% of the total sink strength.

Several other sinks exist in the unirradiated microstructure of ferritic-martensitic steels, such as coarse chromium M_23_C_6_-type carbides and fine V,Cr-nitrides. From previous characterization of T91 heat 30176 used in this work, preexisting precipitates had an estimated number density between 10^18^ and 10^19^ m^-3^ from Section 2.1 above. Radiation-induced Ni/Si clusters that serve as precursors for G-phase precipitates were analyzed in dual ion–irradiated T91 at 445°C, 460°C, 520°C, and 570°C in previous work ^7^ at about 17 dpa. The density of these clusters was found to be about 1 × 10^20^ m^-3^ or less. The preexisting precipitates and radiation-induced clusters have a negligible sink strength compared with dislocation loops and cavities, and therefore they were neglected from the total sink strength calculation for both steady state analysis and CD simulations of dual ion–irradiated T91. Irradiation in the BOR-60 reactor produced a non-negligible concentration of Ni/Si clusters. These clusters were included in the total sink strength as a neutral sink using Eq. (A.3).

- 1. Observation of Helium Trapping Sites

In the main article, we propose an enhanced nucleation process based on helium trapping that has a greater effect on cavity nucleation in reactor irradiation than dual ion irradiation. While this mechanism cannot be viewed directly with TEM, evidence for it can be seen in the local arrangement of cavities, an example of which is in Figure A.11, showing cavities observed in the vicinity of a near edge on the dislocation line.


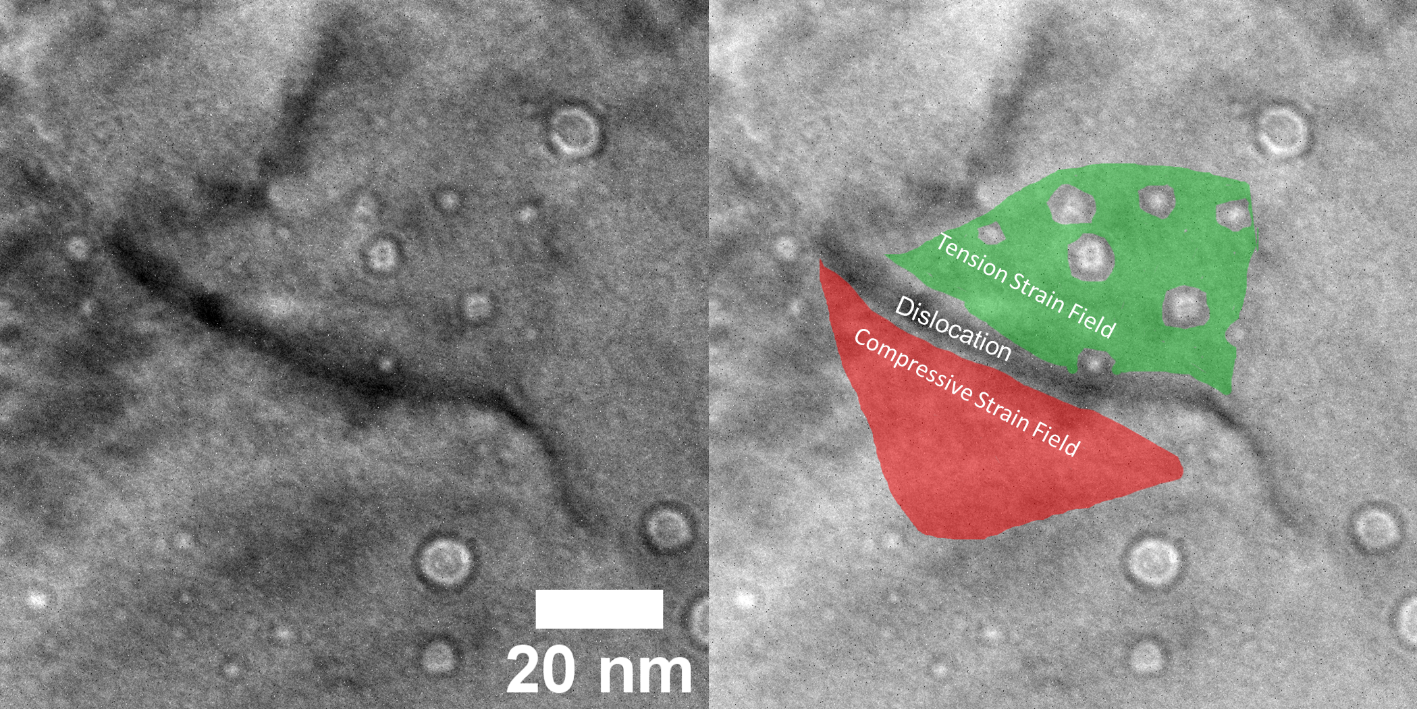


Figure A.11. CTEM BF underfocused micrographs at +2 µm focus on a T91 specimen irradiated to 16.6 dpa at 445°C with 4 appm He/dpa and 7 × 10^-4^ dpa/s. The original image (left) shows cavities in the irradiated microstructure with likely strain fields highlighted (right).

On one side of the cavity, presumed to be the tensile strain field, there is a noticeable increase in the cavity density compared with the other side, inferred to be the compressive strain field. The strain plumes extend an estimated 30–40 nm from the dislocation core and are consistent with the spatially resolved strain fields calculated for dislocations in pure iron in Ref. ^17^ to enhance the vacancy concentration and helium binding energies in the strain fields of dislocations from molecular statics calculations ^18,19^ to trap helium. This variation in the microstructure can lead to net cavity growth in conditions where it would be absent otherwise.

1. **Additional Description of Cluster Dynamics Simulations**

Existing CD work attempting to compensate for dose rate has been confined to very low temperatures and doses due to memory limitations imposed by the large number of equations necessitated by the treatment of three clustering species (interstitials, vacancies, and helium gas atoms) ^20–24^. This work reduces these to a computationally manageable level using a moment-based scheme that groups larger clusters such that the concentration of defects is conserved ^25,26^, allowing the high-dose swelling behavior to be assessed. Even with the grouping scheme in place, the helium-vacancy space is very large such that a robust treatment of dislocation loops and their glide results in a model which is too memory intensive. Instead, loop nucleation is replaced by a single three-dimensionally mobile interstitial equation and a fixed dislocation network. The model was constructed to encompass a full, two-dimensional CD phase space at small sizes where nucleation occurs which is interfaced to a coarse mean size approximation when clusters grow beyond the upper bound. If the exchange between these regimes is sufficiently dynamic and group sizes are kept small, these maintain defect conservation and produce identical results to well-behaved cases employing a full phase space (Figure A.12).


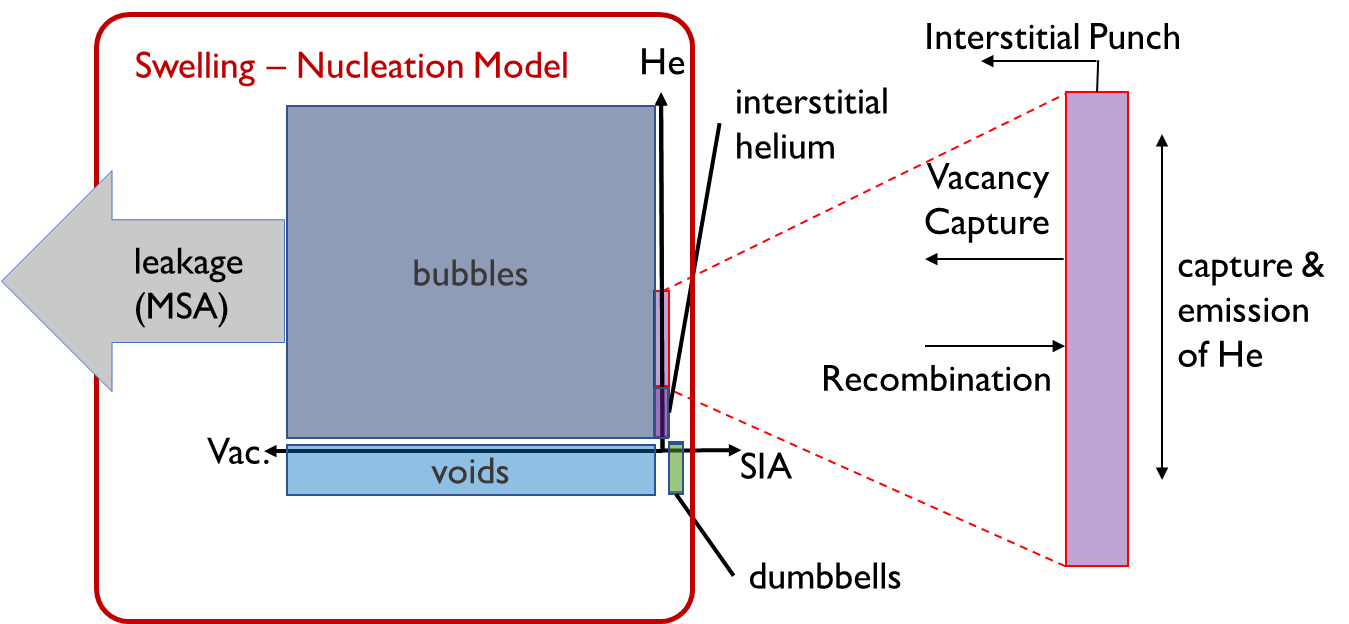


Figure A.12. Visual representation of the phase space of cluster equations considered with respect to defect and helium component axes with the addition of a helium trapping process as a third axis.

- 1. Aggregation and Dissociation Reactions

The nucleation and growth of visible cavity microstructure are the result of a series of individual defect capture events by smaller clusters, represented by the positive component of the interaction term *R* in Eq. (6) in the main article. The defining features of the kinetics in this system are the rate constants *k_j,n_*, which define the rate at which clusters of size *j* and *n* coalesce to form a new cluster (*j+n*). Presuming spherical symmetry and isotropic diffusion, the classical sink strength formulation can be generalized as

|  | $k_{j,n}^{+}=4\pi r_{j,n}(D_{j}+D_{n})$, | (A.6) |
| --- | --- | --- |

where *r_j,n_* is the reaction distance and *D_j_* and *D_n_* are the diffusivities of each species, which are calculated as a function of migration energy *E_m_* and an exponential prefactor *D_0_*:

|  | $D_{j}=D_{0,j}\exp\left(\frac{-E_{m,j}}{k_{b}T}\right)$. | (A.7) |
| --- | --- | --- |

The countervailing process to aggregation at sufficiently high temperatures is thermal emission of single vacancies. For purposes of cluster accounting, each event eliminates one parent cluster and produces one daughter and a mobile monomer according to Eq. (7) in the main article. The rate constant for these reactions is fundamentally a function of how strongly bound each monomer is. Due to the mean field nature of these models, determination is not quite as simple as a treatment mirroring simple diffusion, where an emitted monomer must overcome an activation barrier with some attempt frequency. However, the emitted defect is strongly spatially correlated to its parent cluster, and there is a high likelihood of rapid reabsorption.

To account for this in a mean field sense requires a thermodynamic approach. Because each dissociation reaction has a reverse aggregation reaction, we can build on the assumption that these two are at an equilibrium condition. If the total change in free energy between a bound and unbound state is presumed to be dominated by the binding energy, *E*_b_, the resulting rate constant is

|  | $k_{(j+n)}^{-}=\frac{k_{j,n}^{+}}{\Omega}\exp\left(\frac{-E_{\mathrm{b},(j+n)}}{k_{b}T}\right)$. | (A.8) |
| --- | --- | --- |

- 1. Treatment of Sinks

The conventional calculation of sink strength is performed by defining a cell of appropriate geometry around a sink (spherical for voids, cylindrical for dislocations, etc.) and assuming a steady state solution to the diffusion therein ^27–31^. For the purposes of this work, cavity sink strengths are an intrinsic part of the cluster space and their absorption will be treated individually as part of the larger reaction network. The only external, static sink of major import is therefore the dislocation network, which will serve as the consistent source of bias driving void growth. The analytical solution to the diffusion equation in a cylindrical cell and generalizing to all dislocations in the system yields the sink strength ^32^,

|  | $k_{i}^{\text{disl}}=\frac{2\pi\rho}{\log\left(\frac{1}{\sqrt{\pi\rho r_{c}^{2}}}\right)}$, | (A.9) |
| --- | --- | --- |

where *r_c_* is the dislocation’s capture radius, and ρ is the dislocation density.

Elastic interactions between point defects and nucleating voids were treated by applying a size-independent extension (*r*_ext,_*_j,n_* ) to the geometric radii of each reacting species in the reaction radius used in Eq. (A.6):

|  | $r_{j,n}=r_{\text{geom},j}+r_{\text{geom},n}+r_{\text{ext},j,n}$. | (A.10) |
| --- | --- | --- |

Molecular statics show that these elastic extensions are larger for interstitials than for vacancies, creating a net bias on cavities. This creates an inhibition to nucleation which, unlike thermal emission, cannot be overcome by simply shifting to a lower temperature. The magnitude of these elastic extensions is approximately independent of cavity size, so as cavity size increases, the relative increase in capture volume decays as its contribution relative to the geometric volume is reduced according to

|  | $Z=\frac{k_{i}-k_{v}}{k_{v}}=\frac{r_{\text{ext,i}}-r_{\text{ext,v}}}{r_{\text{void}}+r_{\text{ext,v}}+r_{\text{defect}}}$, | (A.11) |
| --- | --- | --- |

where *Z* is the net bias and *k* is the reaction rate constant given in Eq. (A.6). The result as a functional form, shown in Figure A.13, creates a strong impediment to nucleation. Statics calculations in bcc iron reproducing the work of Kohnert^33^ specify the size of these elastic reaction extensions as 0.32 nm for interstitials and 0.08 nm for vacancies.

0.14

0.65

3.03

14.1

Void Radius [nm]

Figure A.13. Net bias toward interstitial capture on a cavity as a function of its size at several temperatures reproduced using the methodology in Ref. ^33^.

**Additional References**

1. Golubov, S. I., Barashev, A. V. & Stoller, R. E. *Radiation damage theory*. *Comprehensive Nuclear Materials* vol. 1 (Elsevier Inc., 2012).

2. WAS, G. S. *Fundamentals of Radiation Materials Science*. *Fundamentals of Radiation Materials Science* (Springer New York, 2017). doi:10.1007/978-1-4939-3438-6.

3. Greenwood, L. R. & Smither, R. K. *SPECTER: Neutron Damage Calculations for Materials Irradiations*. (1985).

4. Ziegler, J. F., Ziegler, M. D. & Biersack, J. P. SRIM - The stopping and range of ions in matter (2010). *Nuclear Instruments and Methods in Physics Research, Section B: Beam Interactions with Materials and Atoms* vol. 268 1818–1823 (2010).

5. Xu, D., Wirth, B. D., Li, M. & Kirk, M. A. Combining in situ transmission electron microscopy irradiation experiments with cluster dynamics modeling to study nanoscale defect agglomeration in structural metals. *Acta Mater.* **60**, 4286–4302 (2012).

6. Jiao, Z. *et al.* Microstructure evolution of T91 irradiated in the BOR60 fast reactor. *J. Nucl. Mater.* **504**, 122–134 (2018).

7. Taller, S., Jiao, Z., Field, K. & Was, G. S. Emulation of fast reactor irradiated T91 using dual ion beam irradiation. *J. Nucl. Mater.* **527**, 151831 (2019).

8. Taller, S. & Was, G. S. Understanding bubble and void nucleation in dual ion irradiated T91 steel using single parameter experiments. *Acta Mater.* **198**, 47–60 (2020).

9. Parish, C. M. *et al.* LAMDA: Irradiated-Materials Microscopy at Oak Ridge National Laboratory. *Microsc. Microanal.* **21**, 1003–1004 (2015).

10. Parish, C. M., Field, K. G., Certain, A. G. & Wharry, J. P. Application of STEM characterization for investigating radiation effects in BCC Fe-based alloys. *J. Mater. Res.* **30**, 1275–1289 (2015).

11. Phillips, P. J., Brandes, M. C., Mills, M. J. & de Graef, M. Diffraction contrast STEM of dislocations: Imaging and simulations. *Ultramicroscopy* **111**, 1483–1487 (2011).

12. Schindelin, J. *et al.* Fiji: An open-source platform for biological-image analysis. *Nat. Methods* **9**, 676–682 (2012).

13. Zinkle, S. J. & Snead, L. L. Opportunities and limitations for ion beams in radiation effects studies: Bridging critical gaps between charged particle and neutron irradiations. *Scr. Mater.* **143**, 154–160 (2018).

14. Getto, E., Jiao, Z., Monterrosa, A. M., Sun, K. & Was, G. S. Effect of pre-implanted helium on void swelling evolution in self-ion irradiated HT9. *J. Nucl. Mater.* **462**, 458–469 (2015).

15. Field, K. G. *et al.* Dependence on grain boundary structure of radiation induced segregation in a 9 wt.% Cr model ferritic/martensitic steel. *J. Nucl. Mater.* **435**, 172–180 (2013).

16. Duh, T. S., Kai, J. J., Chen, F. R. & Wang, L. H. Effects of grain boundary misorientation on the solute segregation in austenitic stainless steels. *J. Nucl. Mater.* **258**–**263**, 2064–2068 (1998).

17. Kohnert, A. A. & Capolungo, L. A novel approach to quantifying the kinetics of point defect absorption at dislocations. *J. Mech. Phys. Solids* **122**, 98–115 (2019).

18. Heinisch, H. L., Gao, F. & Kurtz, R. J. Atomistic modeling of helium interacting with screw dislocations in α-Fe. *J. Nucl. Mater.* **367**-**370 A**, 311–315 (2007).

19. Heinisch, H. L., Gao, F., Kurtz, R. J. & Le, E. A. Interaction of helium atoms with edge dislocations in α-Fe. *J. Nucl. Mater.* **351**, 141–148 (2006).

20. Kohnert, A. A. & Wirth, B. D. Cluster dynamics models of irradiation damage accumulation in ferritic iron. II. Effects of reaction dimensionality. *J. Appl. Phys.* **117**, 154306 (2015).

21. Kohnert, A. A. & Wirth, B. D. Cluster dynamics models of irradiation damage accumulation in ferritic iron. I. Trap mediated interstitial cluster diffusion. *J. Appl. Phys.* **117**, 154305 (2015).

22. Dunn, A. Y., Capolungo, L., Martinez, E. & Cherkaoui, M. Spatially resolved stochastic cluster dynamics for radiation damage evolution in nanostructured metals. *J. Nucl. Mater.* **443**, 128–139 (2013).

23. Dunn, A., Muntifering, B., Dingreville, R., Hattar, K. & Capolungo, L. Displacement rate and temperature equivalence in stochastic cluster dynamics simulations of irradiated pure α-Fe. *J. Nucl. Mater.* **480**, 129–137 (2016).

24. Xu, D., Vancoevering, G. & Wirth, B. D. Defect microstructural equivalence in molybdenum under different irradiation conditions at low temperatures and low doses. *Comput. Mater. Sci.* **114**, 47–53 (2016).

25. Golubov, S. I., Ovcharenko, A. M., Barashev, A. V. & Singh, B. N. Grouping method for the approximate solution of a kinetic equation describing the evolution of point-defects.

26. Kohnert, A. A. & Wirth, B. D. Grouping techniques for large-scale cluster dynamics simulations of reaction diffusion processes. *Model. Simul. Mater. Sci. Eng.* **25**, (2017).

27. Brailsford, A. D. & Bullough, R. The rate theory of swelling due to void growth in irradiated metals. *J. Nucl. Mater.* **44**, 121–135 (1972).

28. Bullough, R. & Ghoniem, N. M. The Effect of Void Surface Motion on the Void Sink Strength for Point Defects. *J. Nucl. Mater.* **127**, 47–55 (1985).

29. Brailsford, A. D., Bullough, R. & Hayns, M. R. Point defect sink strengths and void-swelling. *J. Nucl. Mater.* **60**, 246–256 (1976).

30. Bullough, R., Hayns, M. R. & Wood, M. H. Sink strengths for thin film surfaces and grain boundaries. *J. Nucl. Mater.* **90**, 44–59 (1980).

31. Bullough, R. & Newman, R. C. The kinetics of migration of point defects to dislocations. *Reports Prog. Phys.* **33**, 101–148 (2002).

32. Trinkaus, H., Naundorf, V., Singh, B. N. & Woo, C. H. On the experimental determination of the migrating defect fraction under cascade damage conditions. *J. Nucl. Mater.* **210**, 244–253 (1994).

33. Kohnert, A. A., Cusentino, M. A. & Wirth, B. D. Molecular statics calculations of the biases and point defect capture volumes of small cavities. *J. Nucl. Mater.* **499**, 480–489 (2018).

1. * Corresponding author: Stephen Taller, P.O. Box 2008, Oak Ridge, TN, 37831

   Notice: This manuscript has been authored by UT-Battelle, LLC, under contract DE-AC05-00OR22725 with the US Department of Energy (DOE). The US government retains and the publisher, by accepting the article for publication, acknowledges that the US government retains a nonexclusive, paid-up, irrevocable, worldwide license to publish or reproduce the published form of this manuscript, or allow others to do so, for US government purposes. DOE will provide public access to these results of federally sponsored research in accordance with the DOE Public Access Plan (<http://energy.gov/downloads/doe-public-access-plan>). [↑](#footnote-ref-2)
